# Self-Guided Beta-Responsive Visual Neurofeedback in a Public Exhibition: Feasibility and Usability Randomized Controlled rial

**DOI:** 10.2196/89256

**Published:** 2026-11-03

**Authors:** Melanie Baumgartner, Raphael Weibel, Tobias Hoesli, Florian von Wangenheim, Joseph Ollier

**Affiliations:** 1 Mobiliar Lab for Analytics, Department of Management, Technology, and Economics, ETH Zurich, Weinbergstrasse 58, Zurich, 8006, Switzerland, +41 44 632 86 24

**Keywords:** neurofeedback, brain-computer interfaces, electroencephalography, beta band activity, mobile health, usability, human-computer interaction

## Abstract

**Background:**

Neurofeedback is studied for stress and anxiety regulation, but most systems require supervision or research-grade equipment. Consumer electroencephalography (EEG) could broaden access, yet autonomous first-contact feasibility, usability, and baseline neural-state effects remain unclear.

**Objective:**

We assessed autonomous completion and usability, tested whether a fixed breathing pacer improved outcomes over visual neurofeedback alone, and explored whether pretask resting beta was associated with usability.

**Methods:**

We conducted a single-session, 2-arm randomized feasibility and usability trial at a public science exhibition in Bern, Switzerland, between March and June 2025. Eligible adults were aged 18 years or older, could read German or French, and reported no epilepsy or implanted electronic device. A tablet app guided participants without experimenter assistance through consent, setup, a 30-second eyes-closed baseline, 1:1 randomization, a 120-second beta-responsive task, and questionnaires. The final analytic sample included participants assigned to visual neurofeedback only (VN; n=27) or visual neurofeedback plus a 6 breaths/minute breathing pacer (VN+BP; n=25). Outcomes were usability, relaxation, and state mindfulness. Data were collected prospectively; no secondary data were used. Analyses used Mann-Whitney *U*, chi-square, Spearman, and permutation tests (2-sided, α=.05); EEG outcome and item analyses were exploratory.

**Results:**

Of 110 visitors initiating a session, 79 completed the task with a valid EEG recording (71.8%, 95% CI 62.8-79.4) and 52 met analytic criteria (47.3%, 95% CI 38.2%-56.5%). Participants (35 women) had a mean age of 39.0 (SD 11.3) years. Usability was good (System Usability Scale [SUS] mean 72.3, SD 14.7; 95% CI 68.3-76.2), and 31/52 participants scored at least 68 (59.6%, 95% CI 46.1%-71.8%). Usability did not differ between VN and VN+BP (median 75.0, IQR 62.5-81.3 vs median 72.5, IQR 65.0-90.0; Mann-Whitney *U*=362.5, *P*=.65; rank-biserial *r*=–0.07, 95% CI –0.39 to 0.25). Relaxation (median 63.0, IQR 50.0-80.0 vs median 61.0, IQR 50.0-67.0; Mann-Whitney *U*=276.5, *P*=.27; *r*=0.18, 95% CI –0.14 to 0.49) and mindfulness (median 2.4, IQR 2.1-2.8 vs median 2.5, IQR 2.0-3.2; Mann-Whitney *U*=346.5, *P*=.68; *r*=–0.07, 95% CI –0.39 to 0.26) did not differ. Higher resting beta was associated with lower SUS before correction (Spearman ρ=–0.31, 95% CI –0.54 to –0.04; raw *P*=.03), but not after Benjamini-Hochberg correction (adjusted *P*=.30). SUS item analyses showed a global pattern (permutation *P*=.02), strongest for functional integration (ρ=–0.43, 95% CI –0.63 to –0.18; Holm-adjusted *P*=.01).

**Conclusions:**

This trial evaluated an autonomous consumer-EEG neurofeedback system integrating self-fitting, signal-quality checking, baseline calibration, adaptive feedback, randomization, and self-report without operator support. Many first-time users completed the autonomous workflow with acceptable-to-good usability; a fixed, open-loop breathing pacer did not improve usability, relaxation, or state mindfulness; and higher resting beta showed a preliminary association with lower perceived usability. These findings support autonomous consumer-EEG neurofeedback and motivate work on adaptive onboarding, validated EEG pipelines, and objective self-regulation outcomes.

## Introduction

Neurofeedback (NFB) trains individuals to change their own brain activity by transforming ongoing neural signals into immediate visual, auditory, or haptic feedback [1]. In classical self-regulation models, such closed-loop systems increase perceived control and self-efficacy because users receive immediate feedback on their internal states and experience that they are capable of voluntarily changing their states and activities [2]. From an embodied and interoceptive perspective, regulation emerges when neural processes, bodily signals, and environmental surroundings are aligned such that internal states become sufficiently explicit for the user to act on them [3]. In other words, effective self-regulation depends on individuals being able to monitor their physiological state effectively [4,5]. For autonomously delivered consumer-EEG (electroencephalography), where expert assistance is unavailable, usability is a prerequisite for successful intervention delivery: users must be able to fit the headset, establish acceptable signal quality, understand the feedback, and complete the task without expert support.

Extant research has shown that NFB can enhance attention, emotion regulation, and stress management in both clinical and nonclinical populations [6,7]. In particular, training to reduce fast neural activity has shown benefits for individuals affected by anxiety symptoms, tension, and depressive symptoms, conditions which are highly prevalent among the general public [8-11]. EEG-based protocols have been used adjunctively in posttraumatic stress disorder (PTSD) and stress-related conditions to stabilize arousal, and performance-oriented NFB has improved affect regulation and cognitive control in nonclinical users [12-14]. A typical target in NFB is oscillatory band modulation. Alpha rhythms (8‐13  Hz) are typically associated with relaxed, inward-focused attention, whereas beta rhythms (13‐30  Hz) are linked to cortical activation, arousal, vigilance, and cognitive effort [8,15-17]. Repeated training can shift these rhythms in desired directions and has been associated with gains in attention, affect regulation, and cognitive performance [14]. Of particular relevance for stress and anxiety disorders is excessive beta activity. Elevated beta power has been associated with hyperarousal and anxious states, and beta downtraining has been reported to reduce tension and improve emotional stability [18-20]. This makes beta-responsive NFB training a theoretically grounded target for supporting self-regulation under stress. Most NFB is still delivered in controlled laboratory or clinical settings with research-grade EEG and expert support [1,21]. Commercial and app-based systems offer a potentially more scalable mode of delivery. Platforms such as Myndlift (Myndlift, Inc) provide remote home NFB with therapist supervision [12], while consumer-grade headsets such as Muse make mobile deployment technically feasible [22-24]. However, these approaches commonly rely on repeated sessions, human guidance, or remote supervision, and evidence on the autonomous first-contact use of consumer-EEG NFB remains limited. This is important because technical availability alone does not ensure successful independent use: without expert support, users must fit the headset, establish acceptable signal quality, understand the feedback, and remain engaged throughout the task [5,25-27]. In addition, little is known about whether users’ pretask neural state is associated with how they experience and evaluate an autonomous NFB system.

We therefore evaluated a fully autonomous, tablet-based, beta-responsive visual NFB system using a consumer EEG headband in a public first-contact setting. The app guided participants through headset fitting, signal-quality checks, baseline recording, an NFB task, and postsession questionnaires without experimenter assistance. Participants received either visual NFB alone or visual NFB with an additional fixed paced-breathing guide. Paced breathing at approximately 6 breaths/minute has been associated with increased heart rate variability, greater parasympathetic influence, and improved baroreflex function [28-30]. The randomized comparison therefore allowed us to test whether adding this open-loop breathing cue improved usability or subjective experience beyond visual NFB alone.

Perceived usability was assessed using the System Usability Scale (SUS) [31,32]. Drawing on physiological computing and neuroadaptive human-computer interaction, we additionally examined pretask neural activity as a potential contextual factor associated with how users experienced the system [5,26,27,33]. Resting beta activity was selected as an exploratory feature because tonic beta has been associated with activation, vigilance, and maintenance of the current cognitive state [15,17]. Examining its relationship with first-contact usability may generate hypotheses for future state-sensitive onboarding approaches, such as adapting pacing, instruction density, or feedback support to the user’s initial state [25,33]

Accordingly, the study addressed three research questions:

1. To what extent can first-contact users successfully complete a fully autonomous NFB workflow in a public setting, and how usable do they perceive the system to be?
2. Does adding a fixed paced-breathing guide to visual NFB affect perceived usability, subjective relaxation, state mindfulness, or descriptive EEG band-power estimates?
3. Is pretask resting beta activity associated with perceived usability in this autonomous first-contact setting?

## Methods

### Trial Design

We conducted a single-session, 2-arm parallel-group randomized feasibility and usability trial with a 1:1 allocation ratio at a public science exhibition in Bern, Switzerland, between March and June 2025. The trial evaluated a fully autonomous, tablet-based, beta-responsive visual NFB system using a consumer EEG headband. The study took place in a semi-enclosed booth that provided a seated, low-distraction environment separated from the main visitor area. The setting was chosen to approximate first-contact, low-support use outside a controlled laboratory or clinical environment. All interactions with the system were designed to be autonomous: participants received instructions, provided consent, completed EEG setup, performed the NFB task, and completed questionnaires without experimenter assistance. No changes to the trial design, eligibility criteria, interventions, or outcome measures were made after trial commencement.

### Reporting Guidelines

This trial is reported in accordance with the CONSORT (Consolidated Standards of Reporting Trials) 2025 statement [34], the CONSORT extension for randomized pilot and feasibility trials [35], and the CONSORT-EHEALTH checklist for reports of randomized trials of web-based and mobile health interventions [36]. The intervention is described in accordance with the TIDieR (Template for Intervention Description and Replication) checklist and guide [37]. The completed checklists are provided in Checklist 1–4.

### Patient and Public Involvement

Patients and members of the public were not involved in the design, conduct, analysis, or reporting of this study beyond their participation as research participants.

### Participants

Participants were identified and recruited by convenience sampling from visitors attending a public science exhibition in Bern, Switzerland, between March and June 2025. Recruitment occurred through exhibition signage and on-site information describing an opportunity to try a NFB exercise. Participation was voluntary and unpaid. Data were collected in a semi-enclosed booth that provided a seated, low-distraction environment separated from the main exhibition area. Eligibility criteria required participants to be at least 18 years old, able to read and respond in German or French, able to provide informed consent, and to report no history of epilepsy or seizures and no implanted electronic medical device, such as a pacemaker or defibrillator. No prior experience with EEG, NFB, meditation, or related technologies was required.

Before entering the study, participants viewed an on-screen information sheet describing the study purpose, procedures, eligibility requirements, potential minor risks, voluntary participation, the right to withdraw, and data handling. They provided digital informed consent through the tablet app by confirming that they understood the study information, met the eligibility criteria, and agreed to participate. For the final analytic sample, records were excluded if participants reported alcohol consumption or stimulant medication use within 8 hours before the session. These exclusions were applied to limit acute physiological confounding and were treated as analytic exclusion criteria rather than safety-related eligibility criteria. Additional data-quality exclusions are described in the “Statistical Methods” section.

### Interventions

The intervention was delivered entirely through a tablet app without direct experimenter assistance. After initiating the session on the tablet, participants watched a 2-minute instructional video explaining the study purpose, safety requirements, eligibility criteria, and procedural steps. They were then guided step by step by on-screen prompts. Participants fitted the Muse 2 consumer EEG headband and adjusted it until the app indicated acceptable contact quality. If acceptable contact quality was not reached, participants could proceed after an on-screen warning; these sessions were flagged by the app, were not recorded, and were excluded from all analyses. Participants then completed a 30-second eyes-closed baseline recording. Immediately after baseline, the app automatically assigned participants in a 1:1 ratio, using simple randomization without blocking or stratification, to visual neurofeedback only (VN) or visual neurofeedback plus breathing pacer (VN+BP). Participants subsequently completed a 120-second beta-responsive visual NFB task in their assigned condition. Before the task, the tablet displayed a short instruction screen with example images of the sky-based feedback. Participants were told that the display would respond to their current brain activity relative to their own baseline. They were encouraged to relax, observe the display, and explore whether they could influence it. The wording avoided explicit claims that the task would necessarily reduce beta activity below the eyes-closed baseline.

After the task, participants removed the headset. The Unity app (Unity Technologies) then directed them to a separate postsession questionnaire in Qualtrics on the same tablet. The questionnaire assessed demographic characteristics, previous NFB-related experience, alcohol consumption, and stimulant medication use within the preceding 8 hours, perceived usability, subjective relaxation, and state mindfulness. Total session time was approximately 10‐12 minutes from consent to completion of the questionnaire. The complete participant workflow was autonomous and conducted on the same tablet without experimenter assistance. Figure 1 illustrates the autonomous experimental workflow.

**Figure 1.**
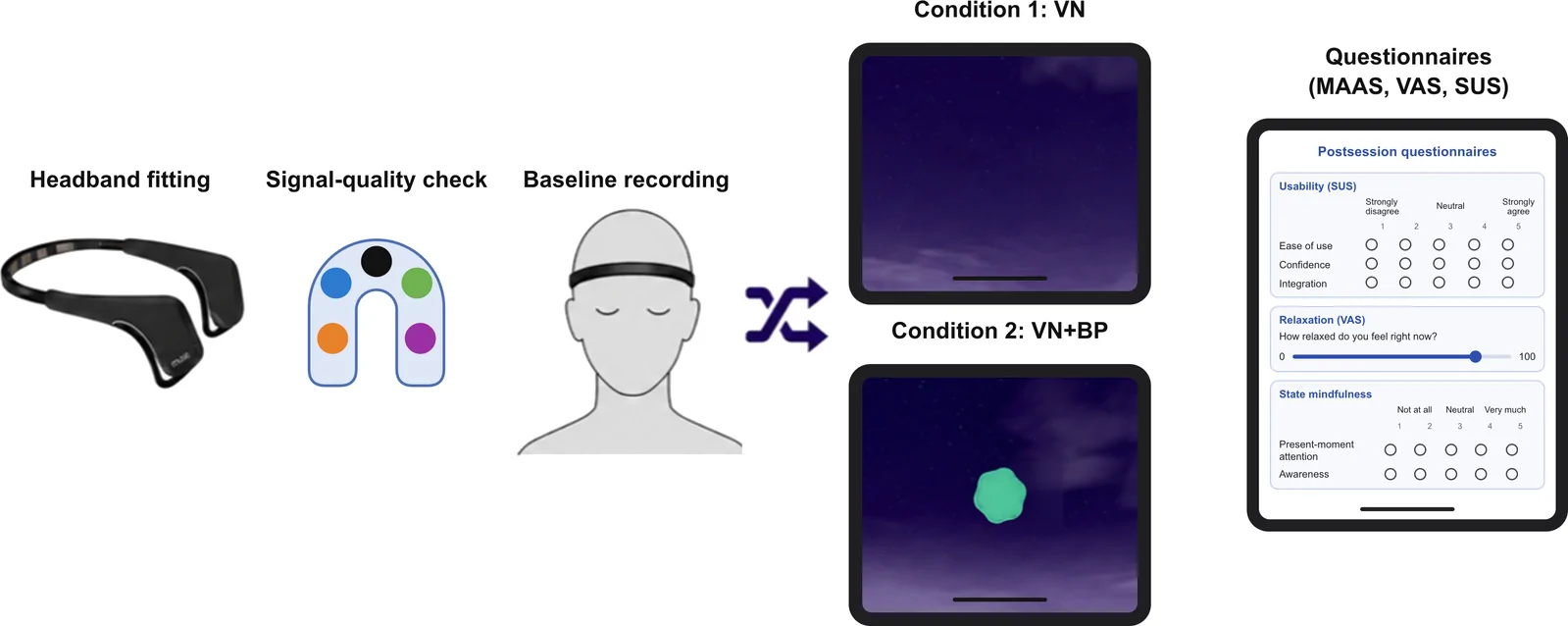
Autonomous experimental workflow of the tablet-guided session, from the instructional video and consent to the postsession questionnaires. MAAS: Mindful Attention Awareness Scale; SUS: System Usability Scale; VAS: Visual Analog Scale; VN: visual neurofeedback only; VN+BP: visual neurofeedback plus breathing pacer.

### NFB System

#### Overview

The NFB system was implemented as a custom Unity app developed using Unity 2022.3.10f1 and integrated with the Muse Unity software development kit (SDK), version 7.2.2. The Unity app handled digital consent and eligibility confirmation, headset setup, signal-quality checking, baseline acquisition, randomized condition assignment, and real-time visual feedback. After completion of the NFB task, the app directed participants to the separate postsession questionnaire in Qualtrics on the same tablet. The visual feedback consisted of a sky-themed environment. The display changed continuously as a function of each participant’s current beta activity relative to their own baseline. Higher relative beta values were mapped to a darker and cloudier sky, whereas values closer to or below baseline were mapped to a clearer sky with greater star visibility. The mapping was continuous and smoothed to avoid abrupt visual changes. If the headset disconnected or the SDK headband-fit status indicated unacceptable signal quality, the display reverted to a neutral intermediate state. The Unity app ran on an Apple iPad with a 10.2-inch display running iPadOS. The tablet was positioned approximately 50 cm in front of the participant at eye level. NFB computation, randomized condition implementation, and display rendering were handled by the Unity app on the tablet. The postsession questionnaire was administered separately through Qualtrics on the same device. The seating position, tablet position, and booth lighting were kept stable across sessions to reduce variation in posture, viewing distance, and visual distraction. For sessions that met the contact-quality threshold, the Unity app stored session metadata, condition assignment, timestamps, SDK-derived band-power values, signal-quality information, and technical events under an anonymous session identifier. The same identifier was entered in Qualtrics, enabling the Unity app and questionnaire records to be linked without using directly identifying information. A short demonstration of the NFB training with the breathing pacer is provided in Multimedia Appendix 1.

#### EEG Acquisition and Feedback Signal

EEG data were acquired using a Muse 2 consumer EEG headband with 4 dry electrodes positioned at AF7, AF8, TP9, and TP10. The Muse Unity SDK provided manufacturer-processed band-power estimates and channel-quality indicators. These SDK-derived values were used for real-time feedback and descriptive EEG analyses. The real-time feedback signal was SDK-derived relative beta power, averaged across AF7, AF8, TP9, and TP10. Relative beta was used for feedback because it expresses beta activity relative to each participant’s concurrent broadband EEG activity, reducing sensitivity to inter-individual differences in absolute signal amplitude, headset fit, and electrode contact. For each participant, the baseline reference was computed as the temporal mean of relative beta during the 30-second eyes-closed baseline.

During the NFB task, the app continuously computed the deviation of current relative beta from the participant-specific baseline:


$$\Delta \beta_{t}=(\beta_{rel,t}-\beta_{baseline})\times 10$$

This value was smoothed in Unity using frame-based linear interpolation:


$${\tilde{\beta }}_{t}=Lerp({\tilde{\beta }}_{t-1},\Delta \beta_{t},0.5\times \Delta t)$$

The resulting smoothed value controlled the visual feedback display. This baseline-referenced relative beta signal was used only for the real-time feedback. For descriptive EEG analyses, we used separately logged SDK-derived absolute band-power estimates, expressed on the manufacturer’s log-scaled arbitrary-unit scale. These absolute band-power estimates did not drive the visual feedback. The SDK-derived estimates were treated as manufacturer-provided features and were not independently validated against an offline EEG preprocessing pipeline. Raw EEG time series were not retained by the acquisition app. The available EEG data therefore consist of SDK-derived band-power streams and associated signal-quality metadata.

#### Experimental Conditions

Participants were randomized to 1 of 2 conditions. In the VN, the screen displayed only the beta-responsive sky environment. Participants were instructed to observe the display, relax, and explore whether they could influence the visual feedback. In the VN+BP, participants saw the same beta-responsive sky environment with an additional centrally overlaid circular breathing guide. The circle expanded during inhalation and contracted during exhalation at a fixed pace of approximately 6 breaths/minute. The breathing pacer was not physiologically closed-loop and did not adapt to EEG or respiratory data. Figure 2 illustrates the beta-responsive visual feedback interface in the VN and VN+BP conditions. The two conditions were otherwise identical in duration, headset setup, baseline procedure, visual environment, questionnaire delivery, and autonomous workflow.

**Figure 2.**
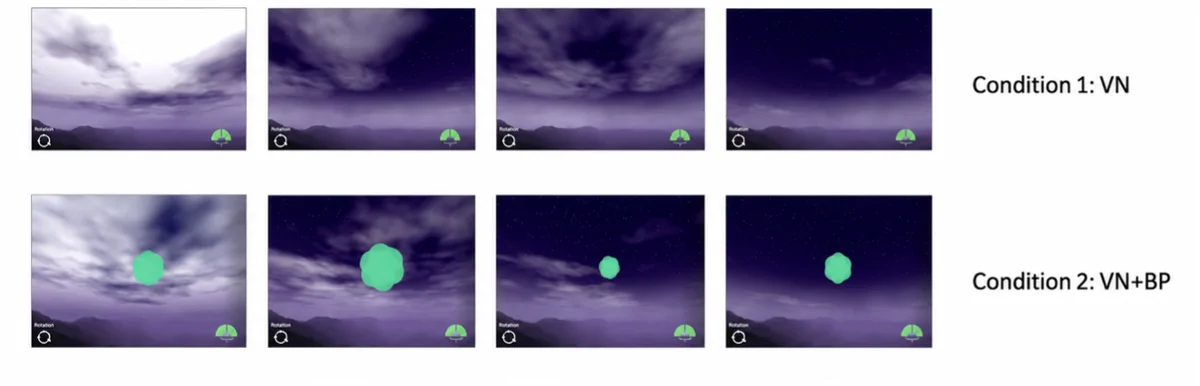
Beta-responsive visual feedback interface in the visual neurofeedback only (VN) and visual neurofeedback plus breathing pacer (VN+BP) conditions.

### Intervention Fidelity

For recorded sessions, the app automatically recorded the anonymous session identifier, condition assignment, phase timestamps, SDK-derived band-power values, signal-quality indicators, and technical events. Completion of the 120-second task was used as the principal measure of intervention completion. The Unity app and Qualtrics questionnaire stored data separately, and records were linked using the anonymous session identifier. Questionnaire responses were not used to control the NFB display or breathing pacer. The intervention was delivered once. No individualized adaptation, additional training, intervention modification, or follow-up session was provided.

### Outcomes

#### Overview

Perceived usability was the primary outcome and was assessed using the 10-item SUS, a widely used instrument for evaluating interactive systems [5,31]. Items are rated on a 5-point scale from 1=strongly disagree to 5=strongly agree. Standard SUS scoring yields a total score from 0 to 100, with higher scores indicating better usability. SUS was analyzed as a continuous variable. For descriptive purposes, scores below 68 were categorized as low usability and scores of 68 or higher as acceptable-or-better usability, following established interpretive guidelines [32]. Subjective relaxation was assessed using a single-item Visual Analog Scale (VAS): “How relaxed did you feel during the neurofeedback session?” Participants responded on a 0‐100 slider, with 0 indicating not at all relaxed and 100 indicating extremely relaxed. State mindfulness was assessed using the state version of the Mindful Attention Awareness Scale–State (MAAS-State) [38]. Items assessed present-moment attention and awareness during the session. After reverse scoring as specified by the scale instructions, higher mean scores indicated greater state mindfulness.

Exploratory EEG features consisted of SDK-derived absolute band-power estimates at baseline and during the task. The main exploratory EEG feature of interest was pretask resting beta power, averaged across AF7, AF8, TP9, and TP10 during the 30-second eyes-closed baseline. Additional descriptive EEG features included theta, alpha, beta, and gamma band-power estimates. Delta-band activity was not included in the main EEG summaries because low-frequency activity in brief, unsupervised consumer EEG recordings with dry electrodes is particularly vulnerable to movement, ocular artifacts, and slow drift. System-performance and feasibility measures included session initiation, task completion, signal-quality failure, setup duration, Bluetooth disconnections, app errors, and data completeness. No formal progression criteria for proceeding to a future definitive randomized trial were prespecified.

#### Sample Size

This trial was designed as an exploratory feasibility and usability study rather than as a confirmatory efficacy trial. The sample size was determined pragmatically by the exhibition period (approximately 3 months), expected visitor flow, and the aim of estimating autonomous completion and perceived usability with reasonable precision. No formal power calculation was performed. Feasibility rates and key usability estimates are therefore reported with 95% CIs, and exploratory EEG-user experience analyses are interpreted cautiously.

### Randomization

#### Sequence Generation

The tablet app generated the condition assignment at the participant level using an internal randomization routine configured for equal allocation to VN and VN+BP. Assignment occurred immediately after completion of the 30-second baseline recording. No participant characteristics were used to determine the assignment. The assigned condition was stored automatically together with the EEG recording.

#### Allocation Concealment Mechanism

Condition assignment was generated and implemented automatically only after completion of the baseline recording. Neither participants nor on-site study personnel had access to the upcoming assignment before allocation. The assigned condition became apparent only after randomization, when the task interface was displayed. No researcher could manually select, alter, or override an assignment.

#### Implementation

Participants initiated and completed the procedure independently. The tablet app enrolled the participant session, generated the allocation, presented the assigned intervention, and recorded the condition. No member of the research team manually generated or communicated assignments.

#### Blinding

Participants could not be blinded to the breathing pacer because it was visually apparent during the task. Both conditions otherwise used the same headset, baseline procedure, beta-responsive sky environment, task duration, general instructions, and postsession questionnaire. No experimenter delivered the intervention or communicated the assigned condition. Questionnaire responses were entered directly by participants through Qualtrics. Analytic exclusion rules were applied without reference to outcome values. Statistical analyses used the recorded condition labels, and no formal analyst-blinding procedure was used.

### Statistical Methods

#### Analysis Populations and Exclusion Criteria

Feasibility analyses used genuine visitor session initiators as the denominator. Sessions involving device testing or kiosk inactivity were not counted as participant sessions. Task-completion analyses included sessions that reached the end of the 120-second NFB task with a valid recording. Sessions that ended before allocation or that proceeded despite insufficient EEG contact quality were included in the feasibility assessment but not in the primary outcome analyses. The analytic sample consisted of participants who completed the NFB task, had a linkable postsession questionnaire record, and met the analytic inclusion criteria. Participants were analyzed according to their randomized condition.

Participants who did not complete the postsession questionnaire were treated as lost to follow-up for the primary outcome. Records were excluded from the primary analytic sample when participants reported alcohol consumption or stimulant medication use during the preceding 8 hours and completed the postsession questionnaire in under 30 seconds.

Participants who reported recent alcohol or stimulant medication use were classified under this exclusion criterion regardless of whether they completed the remaining questionnaire items. Alcohol and stimulant medication questions were administered after the intervention. These responses therefore did not affect eligibility, randomization, or intervention completion. Because allocation could not be retrieved for 17 randomized sessions with insufficient contact quality, and because records were excluded after randomization, the primary outcome analyses were not full intention-to-treat analyses.

#### Missing Data

Participant-level and item-level missingness were assessed separately. Failure to complete the postsession questionnaire was treated as participant-level (unit) nonresponse, that is, as loss to follow-up for the primary outcome. Missing individual responses within otherwise completed questionnaires were treated as item-level missingness. Item-level missingness was assessed across the 10 SUS items, the relaxation VAS, and the 5 MAAS-State items. Derived total and mean scores were not entered alongside their constituent items in the missingness test. A Little missing completely at random (MCAR) test was applied to the 16 raw questionnaire items in the final analytic dataset. Because item-level missingness was minimal, was limited to the secondary MAAS-State outcome, and did not affect the primary SUS outcome, multiple imputation was not performed. Available-case analyses were used, and outcome-specific denominators were reported.

#### Statistical Analyses

Statistical tests were 2-sided with an alpha level of .05. Continuous variables are reported as mean and SD, median and IQR or range, as appropriate. Categorical variables are reported as counts and percentages. Feasibility proportions and other principal proportions were reported with Wilson 95% CIs. Between-condition differences in SUS, subjective relaxation, MAAS-State, and descriptive EEG band-power estimates were examined using Mann-Whitney *U* tests. Rank-biserial correlations and 95% CIs were reported as effect sizes for key continuous comparisons. Categorical variables, including the proportion of participants with SUS scores of at least 68, were compared using chi-square tests or Fisher exact tests when expected cell counts were small. Associations between baseline EEG estimates and subjective outcomes were examined using Spearman rank correlations. The principal exploratory association examined pretask resting beta power and SUS total score. Additional exploratory analyses examined associations between baseline band-power estimates and subjective relaxation and state mindfulness. Partial Spearman correlations adjusted for age and gender were conducted as sensitivity analyses where reported. Multiplicity was addressed separately for the EEG-outcome and SUS item-level analysis families. Benjamini-Hochberg correction was applied across the baseline band-power by subjective-outcome correlation family. For the 10 SUS items, all items were oriented so that higher values indicated better perceived usability. Holm correction and permutation-based family-level tests with 10,000 permutations were used to evaluate the overall item-level pattern and the strongest individual item association.

Additional sensitivity analyses of the beta-usability association included bootstrap and permutation tests of the total-score correlation, leave-one-out influence analyses, robust regression, and item-level modeling. These analyses are reported in Multimedia Appendix 2. EEG-user experience analyses were considered exploratory and hypothesis-generating. Statistical analyses were performed in Python using *NumPy*, *pandas*, *SciPy*, and *statsmodels*. Figures were produced using *matplotlib*. Little MCAR test was performed in R (version 4.6.1; R Core Team) using the mcar_test function from the *naniar* package.

### Ethical Considerations

The trial was reviewed and approved by the ETH Zurich Ethics Commission (reference 25 ETHICS-086). All procedures were conducted in accordance with the Declaration of Helsinki and the institutional guidelines for research with human participants [39]. Before participation, each participant viewed an on-screen information sheet describing the study objective, voluntary participation, the right to withdraw at any time without penalty, data handling, anonymity, minor potential risks such as headband pressure, and eligibility criteria. Participants provided digital informed consent by confirming that they met the eligibility criteria, understood the study information, and agreed to participate. The procedure was available in German or French. No directly identifiable information was collected. Each session was stored under an anonymous session code. Participants received no financial or material compensation. The figures in this paper and the supplementary material do not contain identifiable participant images; the system schematic uses a nonidentifiable illustration.

### Registration

This randomized feasibility and usability trial was not registered prospectively or retrospectively in a primary trial registry. Study materials, deidentified data, and analysis documentation were deposited retrospectively in the Open Science Framework repository.

### Protocol

No prospectively registered trial protocol was available. The Open Science Framework repository contains available study documentation, questionnaire scoring procedures, analytic exclusion rules, deidentified data, and analysis scripts.

## Results

### Participant Flow

#### Overview

A total of 110 visitors initiated an autonomous study session (Figure 3). A total of 14 (12.7%, 95% CI 7.7‐20.2) sessions ended before allocation: 9 during screening and 5 during the baseline recording. The remaining 96 sessions were randomized. In 17 of these (17.7% of randomized sessions, 95% CI 11.4‐26.5), EEG contact quality did not meet the app’s threshold before allocation; participants continued despite an on-screen warning, but these sessions were not recorded and were excluded from all analyses, and their allocation could not be retrieved. Because contact quality was assessed before allocation, these losses are unlikely to be related to the assigned condition. Overall, 79 of 110 (71.8%, 95% CI 62.8‐79.4) session initiators and 79 of 96 (82.3%, 95% CI 73.5‐88.6) randomized participants completed the 120-second NFB task with a valid recording: 42 were allocated to VN and 37 to VN+BP. Among these 79 participants, 4 did not complete the postsession questionnaire and were therefore lost to follow-up for the primary outcome (VN, n=1; VN+BP, n=3). A further 23 participants were excluded from the primary analysis: 21 reported alcohol consumption or stimulant medication use within the preceding 8 hours (VN, n=12; VN+BP, n=9), and 2 completed the questionnaire in under 30 seconds (VN, n=2; VN+BP, n=0). The final analytic sample therefore comprised 52 participants: 27 in VN and 25 in VN+BP. The analytic sample represented 65.8% (52/79) of task completers, 54.2% (52/96) of randomized participants, and 47.3% (52/110; 95% CI 38.2‐56.5) of all initiated sessions. Figure 3 presents the participant flow by randomized condition.

**Figure 3.**
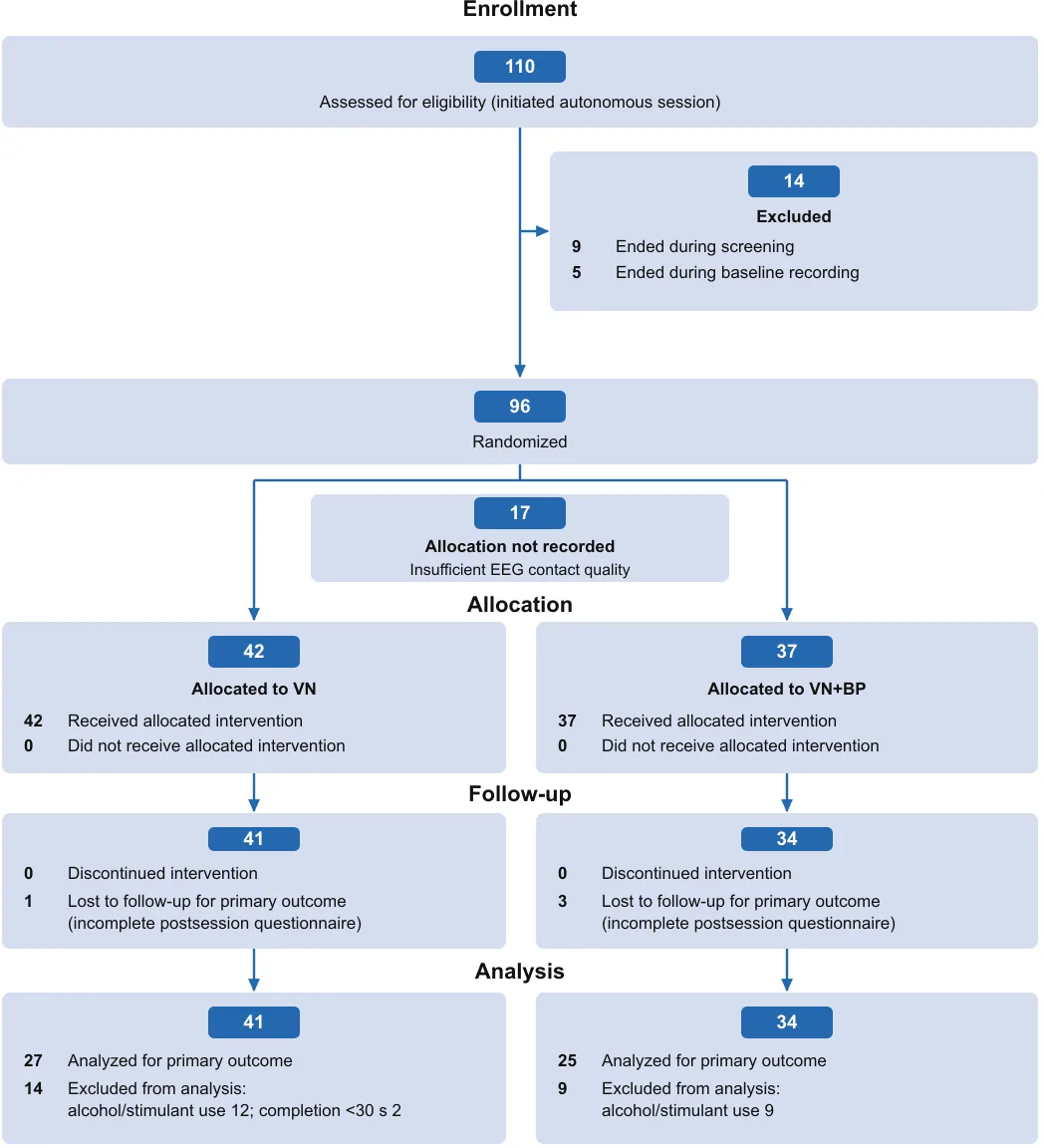
CONSORT (Consolidated Standards of Reporting Trials) 2025 flow diagram of participants through the trial. EEG: electroencephalography; VN: visual neurofeedback only; VN+BP: visual neurofeedback plus breathing pacer.

#### Recruitment

Recruitment and data collection occurred during a public science exhibition in Bern, Switzerland, between March and June 2025. Because the trial involved a single session, there was no follow-up period. The trial ended as planned at the conclusion of the exhibition period.

#### Baseline Data

Participants in the final analytic sample were between 18 and 66 (mean 38.9, SD 11.6; median 37.0, IQR 30.0-47.3) years old; 35 of 52 (67.3%) participants were women. The final analytic sample comprised 27 participants in VN and 25 participants in VN+BP. The groups did not differ in age (Mann-Whitney *U*=329.0, *P*=.84) or gender distribution (*χ*²_₁_=0.002, *P*=.97).

Table 1 summarizes demographic characteristics, system-performance metrics, and data completeness for the final analytic sample.

**Table 1.** Analytic sample characteristics, system performance, and data completeness in a single-session, 2-arm randomized feasibility and usability study of autonomous consumer-EEG (electroencephalography) beta-responsive visual neurofeedback at a public science exhibition in Bern, Switzerland, between March and June 2025^a^.

Characteristic	Total (N=52)	VN^b^ (n=27)	VN+BP^c^ (n=25)	Test statistic	*P* value
Demographics					
Age (years)					
Mean (SD)	38.9 (11.6)	39.1 (11.5)	38.7 (11.9)	329.0^d^	.84
Median (IQR)	37.0 (30.0-47.3)	37.0 (31.0-43.5)	43.0 (30-50)	—^e^	—
Sex, n (%)					
Female	35 (67.3)	18 (66.7)	17 (68.0)	0.002 (1)^f^	.97
Male	17 (32.7)	9 (33.3)	8 (32)	—	—
System performance					
Session duration (min), mean (SD)	11.2 (1.8)	11.0 (1.7)	11.4 (1.9)	318.0^d^	.70
Setup to acceptable signal quality (s), mean (SD)	42.3 (18.6)	40.7 (17.6)	44.1 (19.8)	312.0^d^	.63
Baseline recording duration (s)	30.0 (0.0)	30.0 (0.0)	30.0 (0.0)	—	—
Neurofeedback task duration (s)	120.0 (0.0)	120.0 (0.0)	120.0 (0.0)	—	—
Acceptable EEG signal quality, n (%)	52 (100)	27 (100)	25 (100)	—	—
Bluetooth disconnections, n	0	0	0	—	—
App errors, n	0	0	0	—	—
Data completeness					
Baseline EEG available, n (%)	52 (100)	27 (100)	25 (100)	—	—
Task EEG available, n (%)	52 (100)	27 (100)	25 (100)	—	—
SUS^g^ complete, n (%)	52 (100)	27 (100)	25 (100)	—	—
Relaxation VAS^h^ complete, n (%)	52 (100)	27 (100)	25 (100)	—	—
MAAS-State^i^ score available, n (%)	51 (98.1)	27 (100)	24 (96)	—	—
Overall completeness, n/N (%)	826/832 (99.3)	432/432 (100)	394/400 (98.5)	—	—

a Mann-Whitney *U* tests were used for continuous variables, and chi-square tests were used for categorical variables.

b VN: visual neurofeedback only.

c VN+BP: visual neurofeedback plus breathing pacer.

d Mann-Whitney *U* test value.

e Not applicable.

f Chi-square (*df*) test value.

g SUS: System Usability Scale.

h VAS: Visual Analog Scale.

i MAAS-State: Mindful Attention Awareness Scale–State.

#### Numbers Analyzed

Participant-level and item-level missing data were assessed separately. At the participant level, 31 of the 110 (28.2%) session initiators did not yield a valid task recording: 14 ended before allocation, and 17 proceeded despite insufficient EEG contact quality. Among the 79 task completers with a valid recording, 4 did not complete the postsession questionnaire and were treated as participant-level nonresponse (5.1%). SUS scores, relaxation ratings, baseline EEG estimates, and task EEG estimates were available for all 52 participants in the final analytic sample: 27 in VN and 25 in VN+BP. A MAAS-State score was available for 51 participants: 27 in VN and 24 in VN+BP. One VN+BP participant had no MAAS-State items available and therefore had no score. Another VN+BP participant completed 4 of the 5 MAAS-State items and received a score based on the available items.

Among the 52 records retained in the analytic dataset, all SUS items and relaxation VAS responses were complete. Item-level missingness was confined to the MAAS-State and affected 2 of 52 (3.8%) participants. Six of 832 (0.7%) expected questionnaire-item responses were missing. Little MCAR test provided no evidence against the assumption that item-level missingness was completely at random, *χ*²_26_=15.2, *P*=.95. A sensitivity analysis among these 69 participants yielded the same conclusion, *χ*²_26_=16.0, *P*=.94. The remaining 6 participants excluded from the primary analysis (4 of the 21 excluded for recent alcohol or stimulant use and both participants excluded for a questionnaire duration under 30 seconds) had not provided any responses to the 16 questionnaire items and therefore could not contribute to this test. These results were interpreted cautiously because only 2 records contained missing items, all within the MAAS-State, so the test had limited power to detect departures from MCAR. Multiple imputation was not performed because item-level missingness was minimal and confined to the MAAS-State measure. Outcome-specific available-case analyses were used.

### Outcomes and Estimation

#### Feasibility of Autonomous Use

All 52 retained participants reached acceptable signal quality and completed the baseline and NFB task. Mean setup time from headset placement to acceptable signal quality was 42.3 (SD 18.6) seconds. No Bluetooth disconnections or app errors were recorded in the analytic sample. EEG-derived data, SUS scores, and relaxation ratings were available for all 52 participants, and MAAS-State scores were available for 51 of 52 (98.1%) participants.

#### Psychological Outcomes by Randomized Condition

Table 2 summarizes perceived usability, subjective relaxation, and state mindfulness by randomized condition. Overall usability was good, with a mean SUS score of 72.3 (SD 14.7; 95% CI 68.3‐76.2) and a median score of 75.0 (IQR 63.8-82.5). Using the conventional SUS reference value of 68, 31/52 (59.6%; 95% CI 46.1‐71.8) participants rated the system in the acceptable-or-better usability range. Usability did not differ between VN and VN+BP (median 75.0, IQR 63.8-82.5 vs median 72.5, IQR 65.0-90.0; Mann-Whitney *U*=363.0, *P*=.65; rank-biserial *r*=−0.07, 95% CI −0.39 to 0.25). The proportion of participants with SUS scores of 68 or higher was also similar between conditions (15/25, 60%, vs 16/27, 59.3%; *χ*²_1_=0.002, *P*=.96). The overall median relaxation rating was 62.0 (IQR 50.0-72.3). Relaxation ratings did not differ between VN and VN+BP (median 63.0, IQR 50.0-80.0 vs median 61.0, IQR 50.0-67.0; Mann-Whitney *U*=277.0, *P*=.27; rank-biserial *r*=0.18, 95% CI −0.14 to 0.49). State mindfulness also did not differ between VN and VN+BP (median 2.4, IQR 2.1-2.8 vs median 2.5, IQR 2.0-3.2; Mann-Whitney *U*=346.0, *P*=.68; rank-biserial *r*=−0.07, 95% CI −0.39 to 0.26). Figure 4 visualizes the distributions of perceived usability, subjective relaxation, and state mindfulness by randomized condition.

**Table 2.** Psychological outcomes by randomized condition in a single-session, 2-arm randomized feasibility and usability study of autonomous consumer-EEG (electroencephalography) beta-responsive visual neurofeedback^a^.

Outcome	Total (N=52)	VN^b^ (n=27)	VN+BP^c^ (n=25)	Effect size, *r* (95% CI)	Test statistic	*P* value
SUS^d^ score, mean (SD)	72.3 (14.7)	71.2 (12.6)	73.4 (16.8)	—^e^	—	—
SUS score, median (IQR)	75.0 (63.8-82.5)	75.0 (62.5-81.3)	72.5 (65.0-90.0)	−0.07 (−0.39 to 0.25)^f^	363.0^g^	.65
SUS ≥68, n/N (%)	31/52 (59.6)	16/27 (59.3)	15/25 (60.0)	—	0.002 (1)^h^	.96
Relaxation VAS^i^, mean (SD)	59.4 (18.7)	61.8 (19.7)	56.8 (17.6)	—	—	—
Relaxation VAS, median (IQR)	62.0 (50.0-72.3)	63.0 (50.0-80.0)	61.0 (50.0-67.0)	0.18 (−0.14 to 0.49)^f^	277.0^g^	.27
MAAS-State^j^, mean (SD)	2.5 (0.7)	2.4 (0.5)	2.5 (0.8)	—	—	—
MAAS-State, median (IQR)	2.4 (2.0-2.8)	2.4 (2.1-2.8)	2.5 (2.0-3.2)	−0.07 (−0.39 to 0.26)^f^	346.0^g^	.68

a Mann-Whitney *U* tests were used for continuous or ordinal outcomes; chi-square tests were used for categorical outcomes. MAAS-State scores were available for 51 participants (VN, n=27; VN+BP, n=24). One VN+BP participant completed 4 of the 5 items and received a score based on the available items; another VN+BP participant had no MAAS-State items available and therefore had no score.

b VN: visual neurofeedback only.

c VN+BP: visual neurofeedback plus breathing pacer.

d SUS: System Usability Scale.

e Not applicable.

f Rank-biserial.

g Mann-Whitney *U* test value.

h Chi-square (*df*) test value.

i VAS: Visual Analog Scale.

j MAAS-State: Mindful Attention Awareness Scale–State.

**Figure 4.**
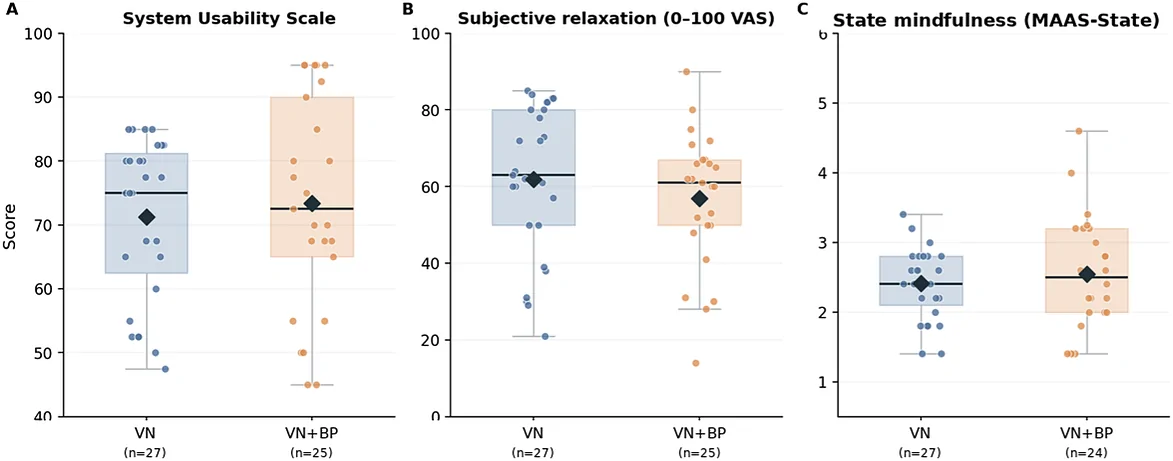
Psychological outcomes by randomized condition. MAAS-State: Mindful Attention Awareness Scale–State; SUS: System Usability Scale; VAS: Visual Analog Scale; VN: visual neurofeedback only; VN+BP: visual neurofeedback plus breathing pacer.

#### Descriptive SDK-Derived EEG Band-Power Estimates

Table 3 reports SDK-derived absolute band-power estimates during the 30-second eyes-closed baseline and the 120-second NFB task. These values are reported descriptively to characterize the EEG feature space in the retained analytic sample. They should not be confused with the real-time feedback signal: the visual display was driven by baseline-referenced relative beta, whereas Table 3 reports separately logged SDK-derived absolute band-power estimates.

**Table 3.** Software development kit–derived absolute electroencephalography band-power estimates at baseline and during the 120-second neurofeedback task^a^.

Band	Baseline total (N=52), mean (SD)	Baseline VN^b^ (n=27), mean (SD)	Baseline VN+BP^c^ (n=25), mean (SD)	Task total (N=52), mean (SD)	Task VN (n=27), mean (SD)	Task VN+BP (n=25), mean (SD)
Theta, 4‐8 Hz	0.39 (0.31)	0.37 (0.29)	0.41 (0.33)	0.42 (0.34)	0.40 (0.32)	0.44 (0.36)
Alpha, 8‐13 Hz	0.51 (0.33)	0.48 (0.31)	0.54 (0.36)	0.48 (0.31)	0.46 (0.29)	0.50 (0.33)
Beta, 13‐30 Hz	0.36 (0.29)	0.34 (0.27)	0.38 (0.31)	0.38 (0.31)	0.36 (0.28)	0.40 (0.34)
Gamma, 30‐50 Hz	0.10 (0.28)	0.09 (0.26)	0.11 (0.30)	0.11 (0.29)	0.10 (0.27)	0.12 (0.31)

a Values are software development kit–derived absolute band-power estimates averaged across AF7, AF8, TP9, and TP10 and expressed on the manufacturer’s log-scaled arbitrary-unit scale. Baseline refers to the 30-second eyes-closed recording; task refers to the 120-second beta-responsive neurofeedback task. These descriptive absolute band-power estimates did not drive the visual feedback display; real-time feedback was driven by baseline-referenced relative beta.

b VN: visual neurofeedback only.

c VN+BP: visual neurofeedback plus breathing pacer.

Across participants, the descriptive spectral profile was similar at baseline and during the task. Mean beta power was 0.36 (SD 0.29) during baseline and 0.38 (SD 0.31) during the task. Between-condition comparisons showed no statistically significant differences in SDK-derived absolute band power for theta, alpha, beta, or gamma at baseline or during the task.

Between-condition comparisons showed no significant differences in absolute band power at baseline (theta: Mann-Whitney *U*=297.0, *P*=.46; alpha: Mann-Whitney *U*=312.0, *P*=.63; beta: Mann-Whitney *U*=289.0, *P*=.37; gamma: Mann-Whitney *U*=301.0, *P*=.50) or during the task (theta: Mann-Whitney *U*=305.0, *P*=.55; alpha: Mann-Whitney *U*=318.0, *P*=.70; beta: Mann-Whitney *U*=294.0, *P*=.42; gamma: Mann-Whitney *U*=308.0, *P*=.58).

### Ancillary Analyses

#### Exploratory Resting Beta-Usability Association

Because between-condition differences were minimal, exploratory individual-difference analyses were collapsed across randomized conditions. Baseline beta power refers to SDK-derived absolute beta-band power during the 30-second eyes-closed baseline, averaged across AF7, AF8, TP9, and TP10 and expressed on the manufacturer’s log-scaled arbitrary-unit scale.

Higher pretask resting beta power was associated with lower SUS total scores in the uncorrected Spearman rank correlation (ρ=−0.31, 95% CI −0.54 to −0.04, raw *P*=.03; Table 4). This total-score association did not survive Benjamini-Hochberg correction across the EEG-outcome family (adjusted *P*=.30). The corresponding Pearson correlation was not statistically significant (*r*=−0.24, *P*=.08). A sensitivity analysis adjusting for age and gender showed a similar negative association (partial Spearman ρ=−0.33, *P*=.02).

Baseline beta power was not significantly associated with subjective relaxation or state mindfulness (Table 4). Figure 5 visualizes the exploratory association between pretask resting beta power and SUS total score.

**Table 4.** Exploratory associations between pretask resting beta power and subjective outcomes^a^.

Analysis	N	Spearman ρ estimate	95% CI	Raw *P* value	Adjusted or permutation *P* value
Baseline beta × SUS^b^ total score	52	−0.31	−0.54 to −0.04	.03	.30^c^
Baseline beta × relaxation VAS^d^	52	−0.13	−0.39 to 0.15	.37	.61^c^
Baseline beta × MAAS-State^e^	51	0.11	−0.17 to 0.37	.44	.61^c^
SUS item 5: functional integration	52	−0.43	−0.63 to −0.18	.001	.01^f^; .02^g^
Global SUS item pattern	52	9/10 item correlations negative	—	—	.02^h^

a Baseline beta refers to software development kit–derived absolute beta-band power during the 30-second eyes-closed baseline, averaged across AF7, AF8, TP9, and TP10. Electroencephalography-outcome analyses were corrected across the baseline band-power by subjective-outcome family. System Usability Scale item analyses used permutation-based family-level tests with 10,000 permutations. All analyses in this table are exploratory.

b SUS: System Usability Scale.

c Benjamini-Hochberg–adjusted.

d VAS: Visual Analog Scale.

e MAAS-State: Mindful Attention Awareness Scale–State.

f Holm-adjusted.

g Max-item permutation.

h Permutation.

**Figure 5.**
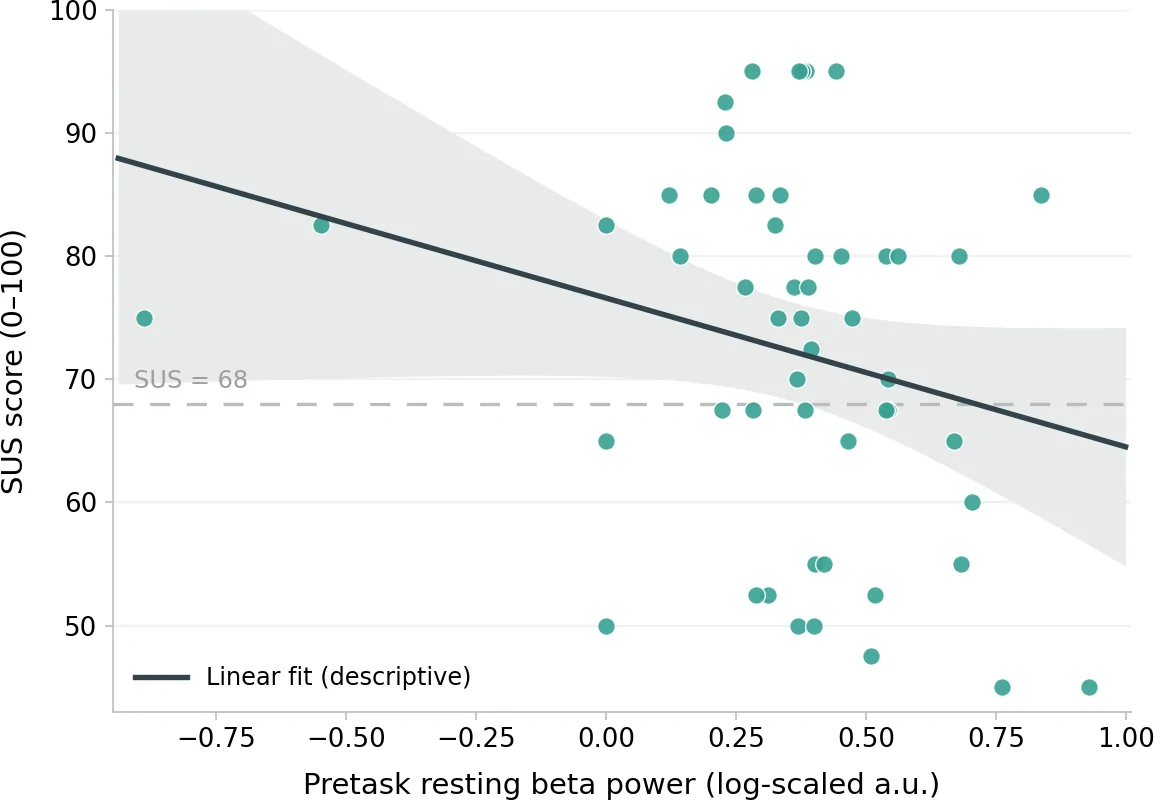
Pretask resting beta power and perceived usability. Scatterplot of software development kit–derived absolute beta-band power during the 30-second eyes-closed baseline and System Usability Scale (SUS) score in the final analytic sample (N=52). Baseline beta power was averaged across AF7, AF8, TP9, and TP10 and is expressed on the manufacturer’s log-scaled arbitrary-unit scale. The dashed horizontal line indicates the conventional SUS reference value of 68. The fitted line is shown for descriptive visualization; inferential testing used Spearman rank correlation. Higher resting beta power was associated with lower SUS scores in the uncorrected Spearman correlation (ρ=−0.31, 95% CI −0.54 to −0.04, raw *P*=.03), but the total-score association did not survive Benjamini-Hochberg correction across the electroencephalography-outcome family (adjusted *P*=.30). Point colors, if used, indicate whether SUS scores were below or above 68 and are shown only for descriptive orientation. The figure should not be interpreted as evidence of prediction, causality, beta suppression, or learned beta regulation.

#### Exploratory SUS Item-Level Analyses

Exploratory SUS item-level analyses were conducted to examine the association pattern across the 10 SUS items. All items were oriented so that higher scores indicated better perceived usability. The family-level permutation test across SUS items was significant (global permutation *P*=.02). The strongest item-level association was observed for SUS item 5, perceived functional integration: higher baseline beta power was associated with lower perceived functional integration (Spearman ρ=−0.43, 95% CI −0.63 to −0.18, raw *P*=.001; Holm-adjusted *P*=.01; max-item permutation *P*=.02).

Figure 6 shows the item-level association pattern, the strongest item-level association for SUS item 5, and a descriptive time-course visualization of SDK-derived beta power across baseline and task by conventional SUS category.

**Figure 6.**
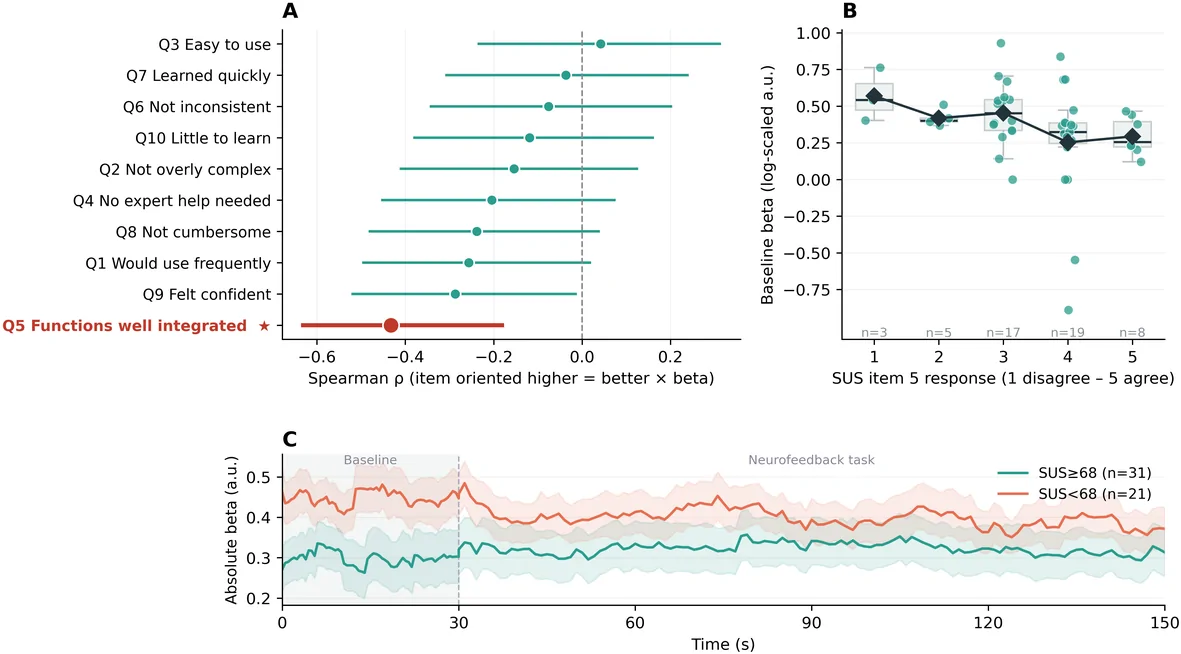
Exploratory System Usability Scale (SUS) item-level associations and descriptive beta time course. (A) Spearman correlations between software development kit (SDK)–derived absolute beta-band power during the 30-second eyes-closed baseline and the 10 SUS items, with all SUS items oriented so that higher scores indicate better perceived usability. Points indicate Spearman ρ estimates and horizontal bars indicate 95% CIs. The dashed vertical line marks ρ=0. SUS item 5, perceived functional integration, is highlighted as the strongest item-level association. (B) SDK-derived absolute beta-band power by response level on SUS item 5, “I found the various functions in this system were well integrated.” Points represent individual participants; boxes show median and IQR; diamonds indicate category means; response counts are shown below each category. The connecting line between category means is shown only for visual orientation. (C) Mean SDK-derived absolute beta-band power across the 30-second eyes-closed baseline and the 120-second neurofeedback task for participants with SUS scores below 68 and participants with SUS scores of 68 or higher. The vertical dashed line indicates the transition from baseline to the neurofeedback task. Shaded regions indicate ±1 SEM. In all panels, beta power was averaged across AF7, AF8, TP9, and TP10 and is expressed on the manufacturer’s log-scaled arbitrary-unit scale. SUS item-level analyses were exploratory. The global item-level permutation test indicated a nonrandom item-level association pattern (global permutation *P*=.02). The strongest item-level association was observed for SUS item 5, perceived functional integration (Spearman ρ=−0.43, 95% CI −0.63 to −0.18, raw *P*=.001), which remained significant after correction across the 10 SUS items (Holm-adjusted *P*=.014; max-item permutation *P*=.02). Panel C uses post hoc SUS categories based on the conventional SUS reference value of 68 and is shown only for descriptive visualization. Panel C shows SDK-derived absolute beta-band estimates, not the baseline-referenced relative beta signal that drove the visual feedback display. The figure should not be interpreted as evidence of prediction, causality, beta suppression, or learned neurofeedback regulation.

#### Harms

Harms and minor transient discomfort were not assessed systematically. This limitation is also relevant to the broader consumer-NFB literature, in which adverse effects have often not been systematically evaluated [7,40]. The absence of reported adverse events in the present trial should therefore not be interpreted as evidence that no discomfort or other unintended effects occurred.

## Discussion

### Interpretation

This study examined 3 questions: whether first-contact users could complete a fully autonomous consumer-EEG NFB workflow in a public setting, whether adding a fixed paced-breathing guide improved usability or subjective experience, and whether pretask resting beta activity was associated with perceived usability. Three main findings emerged. First, autonomous use was feasible for many participants: most session initiators completed the NFB task, and participants retained in the analytic sample rated the system in the acceptable-to-good usability range. At the same time, the full initiation denominator showed that autonomous task completion was feasible for a majority, although not all initiated sessions progressed to a complete analyzable record. Second, adding a fixed, open-loop breathing pacer did not improve perceived usability, subjective relaxation, state mindfulness, or descriptive EEG band-power estimates compared with visual NFB alone. Third, exploratory analyses indicated a negative association between pre-task resting beta activity and perceived usability, with the clearest item-level association observed for perceived functional integration. However, as the total-score association did not remain significant after correction across the broader EEG-outcome family, these neural findings should be interpreted as hypothesis-generating rather than confirmatory.

### Autonomous First-Contact Feasibility and Usability

The principal contribution of this trial was the evaluation of a consumer-EEG NFB system as a complete autonomous first-contact pathway rather than only as a portable feedback interface. NFB research has traditionally relied on structured laboratory or clinical protocols in which trained personnel support EEG setup, signal-quality assessment, task delivery, and interpretation of the feedback [1,41,42]. Remote and consumer-facing approaches have expanded NFB beyond conventional laboratories, but many retain repeated-session structures, remote support, or some degree of professional involvement [12,40]. Consumer EEG hardware has nevertheless made lower-burden and more mobile deployment increasingly feasible, with recent studies demonstrating applications of wearable EEG and Muse-type systems outside conventional research-grade setups [21,43-45]. The present system extended this approach by integrating consent, headset fitting, signal-quality checking, baseline calibration, randomization, real-time feedback, and transition to postsession questionnaires within a workflow that participants completed without direct experimenter assistance. The observed completion pattern provides a more nuanced picture than usability scores alone. Most visitors who initiated the procedure completed the NFB task, suggesting that the workflow was manageable for many first-contact users in a low-support public setting. However, 31 of 110 initiated sessions did not yield a valid task recording. Insufficient EEG contact quality was the most frequent reason (17 sessions), exceeding sessions that ended before allocation (14 sessions), which indicates that achieving adequate electrode contact without assistance was the main barrier in the autonomous pathway. In addition, 4 participants did not complete the postsession questionnaire, and post-task exclusions reflected prespecified analytic and data-quality criteria, namely recent alcohol or stimulant use and implausibly rapid questionnaire completion; these exclusions should be distinguished from failures of the interface itself. This distinction is important for autonomous neurotechnology because feasibility encompasses several stages: initiating the system, establishing usable physiological signals, completing the intervention, and ultimately providing analyzable data. Contemporary brain-computer interface (BCI) and digital-health usability research similarly emphasizes that successful translation depends not only on algorithmic performance but also on setup burden, reliability, usability, satisfaction, and the ability of users to operate sensor-based systems independently [5,25].

Among retained participants, perceived usability fell within the conventionally interpreted acceptable-to-good range, based on established SUS interpretation and more recent digital-health benchmarking [31,32]. Setup was generally brief, data completeness was high, and no major app or connectivity problems were observed in the analytic sample. These findings complement previous research suggesting that consumer EEG can support NFB-related and psychophysiological applications outside specialized laboratories [21,43,44,46]. They also contribute to a growing literature on consumer-grade NFB, although recent meta-analytic evidence emphasizes that successful deployment and favorable user experience should be distinguished from demonstrated modulation of the intended neural target [40]. More broadly, usability and acceptance are established early outcomes in proof-of-concept evaluations of adaptive digital-health systems, while BCI usability research has highlighted workload, satisfaction, reliability, and ease of access as central requirements for practical translation [5,25]. This study extends this work to the technically demanding context of autonomous NFB, in which users must independently fit EEG hardware, establish adequate signal quality, complete baseline calibration, and interpret real-time physiological feedback. For autonomous NFB, usability has a particularly direct functional role. BCI research has emphasized that practical deployment depends not only on signal-processing performance but also on ergonomic factors, sensor setup, reliability, and the overall user experience [5,25,27]. Consumer EEG systems offer greater portability and accessibility, but wearable and dry-electrode recordings remain sensitive to electrode contact, movement, and other sources of signal degradation [43-45]. In supervised settings, an expert can assist with headset positioning, poor signal quality, task comprehension, and technical problems; in an autonomous workflow, these functions must instead be supported by the instructions, interface, and system logic [5,47]. This study therefore contributes evidence about the feasibility of the complete first-contact pathway rather than only the feedback display or the experience of users who had already completed setup. The favorable usability ratings should not, however, be interpreted as evidence of therapeutic benefit or successful NFB learning. This was a brief, single-session study without a sham-feedback, no-feedback, or no-intervention control condition. The results show that many first-time users could operate and evaluate the system autonomously; they do not establish that the task reduced symptoms, improved self-regulation, or produced learned control over beta activity. This distinction is important when positioning the present findings within the broader BCI literature; evidence for therapeutic BCI effects generally comes from repeated, task-specific clinical interventions, such as BCI-supported upper-limb rehabilitation after stroke [27,48]. This study instead evaluated a brief, autonomous first-contact experience in a nonclinical setting and should not be interpreted as a test of therapeutic BCI efficacy.

### No Detectable Added Benefit of Fixed Breathing Guidance

The breathing pacer was included as a simple body-based cue that might support relaxation and make the visual feedback easier to follow. Slow breathing at approximately 6 breaths/minute has been associated with greater heart rate variability, increased parasympathetic influence, and enhanced baroreflex function [29,30]. Breath-focused practices may also influence attention and immediate psychological state, and recent work has shown that slow breathing can modulate EEG [28,49]. It was therefore plausible that adding paced-breathing guidance to the visual NFB task could improve the user experience. In this study, however, the fixed breathing pacer produced no detectable incremental improvement in perceived usability, subjective relaxation, state mindfulness, or descriptive EEG band-power estimates. This finding is specific to the brief, fixed, open-loop breathing overlay tested here and should not be interpreted as evidence that paced breathing is generally ineffective. Slow or resonance-frequency breathing has well-established effects on autonomic cardiovascular dynamics [29,30], and related biofeedback research has demonstrated effects on heart rate variability and user experience [50]. Nor does the present result establish that visual NFB itself caused the favorable subjective outcomes. Because both randomized conditions received the same beta-responsive visual NFB, the comparison isolates only whether the additional breathing guide improved outcomes beyond visual NFB alone.

Several aspects of the implementation may have limited the added value of the breathing guide. The NFB task lasted only 120 seconds, which may have provided insufficient time for users to establish a stable slow-breathing rhythm [50]. The pacer used the same breathing rate for all participants and did not adapt to individual respiration, comfort, EEG activity, or autonomic state. Respiration and heart rate variability were not recorded, so it was not possible to determine whether participants followed the guide or whether it produced the intended physiological response. In addition, the breathing display may have been unnecessary for some participants, while for others it may have increased the amount of visual information competing for attention.

From a physiological-computing perspective, the relevant question may therefore be not simply whether breathing guidance is present, but whether its timing and intensity are appropriate for the individual user [50]. Recent work on human-centered digital health and adaptive closed-loop BCI systems supports greater attention to individualization and context-sensitive adaptation [25,33]. Future research could compare fixed with individualized breathing rates, measure respiratory adherence and autonomic responses, and test whether breathing guidance is more effective when introduced selectively; for example, when a user shows difficulty settling, unstable physiological signals, or reduced engagement. Such adaptive support remains a design hypothesis and was not evaluated in this study. The absence of differences in descriptive EEG measures also reinforces the need to distinguish a beta-responsive feedback task from demonstrated beta downregulation. The system continuously changed the visual environment according to each participant’s baseline-referenced relative beta signal, but the study did not show that participants learned to reduce beta activity. This distinction is consistent with recent meta-analytic evidence showing that the use of consumer-grade NFB does not by itself establish reliable modulation of the intended neural target [40]. Demonstrating NFB learning requires evidence that the targeted neural feature changes systematically through interaction with the feedback, ideally across repeated training and against an appropriate control condition [42,51]. The current contribution lies in autonomous delivery and usability, not in evidence of successful beta modulation.

### Exploratory Resting Beta-Usability Association

The exploratory EEG analyses suggest that the experience of an autonomous NFB interface may depend partly on the user’s state before the task begins. Higher pretask resting beta activity was associated with lower SUS scores in the uncorrected analysis, and the direction of the association remained similar after adjustment for age and gender. However, the total-score association did not survive correction across the broader EEG-outcome family. The result should therefore not be considered confirmatory. The item-level analyses provided a more specific, although still exploratory, pattern. Most item correlations were negative, the overall item-level pattern was unlikely to have arisen by chance, and the clearest association concerned whether the system’s functions felt well integrated. This suggests that resting beta may have been related more closely to the perceived coherence of the interaction than to subjective relaxation or state mindfulness [15]. Nevertheless, the individual item findings should be interpreted cautiously because some response categories contained relatively few observations and the analyses were not preregistered. Independent replication is needed before this pattern can be treated as reliable. Beta-band activity has been associated with cortical activation, vigilance, cognitive effort, and maintenance of the current neural or behavioral state [15,18,52]. Some NFB studies have also examined elevated high-beta activity in relation to anxious or hyperaroused states and have investigated beta downtraining in participants with anxiety or depressive symptoms [8,17]. More recently, resting-state EEG research has reported increased beta-band activity in patients with generalized anxiety disorder compared with healthy controls [20]. However, beta activity is not a specific measure of anxiety, and EEG biomarkers across anxiety and related psychiatric conditions remain heterogeneous [19]. This study did not assess baseline anxiety or clinical symptoms. It would therefore be inappropriate to conclude that participants with higher beta were more anxious, more in need of treatment, or less likely to benefit from NFB. One possible interpretation is that resting beta reflected a pretask neural state that influenced how an unfamiliar interface was experienced [15]. Although this study did not measure this process directly, a user entering the session in a more effortful or evaluative state might experience the relationship between headset setup, baseline calibration, visual feedback, and breathing guidance as less immediately coherent. This interpretation is consistent with physiological-computing and adaptive-BCI frameworks, in which physiological activity can function not only as a feedback target but also as a contextual variable related to interaction demands and user experience [5,26,33]. It remains, however, one possible explanation rather than a mechanism established by the data. If the association is replicated, baseline state could become relevant to the design of autonomous onboarding. Physiological and neural signals have increasingly been considered as potential inputs for adaptive system behavior in BCI and neuroergonomic research [5,27,33], while consumer-NFB research has identified calibration and individualization as priorities for future systems [40]. Building on these approaches, future studies could test whether users entering a session with higher pretask activation benefit from a longer settling phase, reduced instruction density, slower feedback transitions, or additional guidance, whereas users showing lower initial activation could proceed directly to the task. The present findings do not establish resting beta as a usability biomarker; rather, they provide a candidate state variable for prospectively testing such adaptive onboarding strategies. No formal progression criteria were prespecified, and the present trial was not designed to support a formal decision on whether to proceed to a definitive trial. Before a larger confirmatory trial, future studies should prespecify feasibility thresholds, retain and validate raw EEG, assess objective target engagement and breathing adherence, and evaluate repeated use with an appropriate control condition.

### Limitations

This study has several limitations. First, it was a brief, single-session feasibility and usability study conducted at a public science exhibition. Participants were self-selected visitors interested in trying a neurotechnology demonstration and were not representative of the general population or a clinical group. The findings therefore concern autonomous first-contact use and cannot establish repeated-session adherence, NFB learning, durable self-regulation, symptom improvement, or clinical efficacy. Second, the study did not include sham feedback, breathing-only, or no-intervention conditions. Consequently, the observed usability, relaxation, and mindfulness ratings cannot be attributed specifically to NFB. The randomized comparison tested only whether adding the breathing pacer changed outcomes relative to visual NFB alone. Third, EEG was recorded with a 4-channel consumer headband. This enabled a scalable autonomous procedure but limited spatial inference and increased reliance on manufacturer-provided signal processing. The app did not retain raw EEG time series, preventing independent offline artifact removal, spectral estimation, or validation against a research-grade system. Consumer EEG recordings may be affected by differences in electrode contact, eye movements, cranial muscle activity, movement-related artifacts, and device-specific signal-processing characteristics [43-45]. In 17 of 96 randomized sessions, contact quality was insufficient; these sessions were not recorded, and their allocation could not be retrieved. Because contact quality was assessed before allocation, these losses are unlikely to be related to the assigned condition, but they reduced the analyzable sample. The real-time and analytic EEG measures also differed. The display was driven by baseline-referenced relative beta provided by the Muse SDK, whereas the exploratory analyses used separately logged SDK-derived absolute band-power estimates. These signals serve different purposes and should not be treated as equivalent. In addition, the eyes-closed baseline and the visually engaging task differed in visual input and attentional demands, limiting direct interpretation of baseline-to-task band-power differences as NFB-related regulation [53,54]. Fourth, the beta-usability analyses were exploratory and based on a modest sample. The total SUS association did not remain significant after correction across the broader EEG-outcome family, while the item-level result requires replication because it decomposed a composite measure into individual responses. Future studies should preregister the beta-usability hypothesis, define multiplicity families in advance, retain raw EEG, and compare consumer-derived features with validated offline pipelines and research-grade recordings. Fifth, the study relied predominantly on self-report outcomes. The SUS is an established measure of perceived usability [5,31], but it does not assess whether participants understood the feedback accurately, achieved objective control over the target signal, or would continue using the system. Future work should combine perceived usability with behavioral completion measures, comprehension checks, objective NFB-control indices, respiration, heart rate variability, state anxiety, perceived stress, workload, and prior experience with digital or neurotechnology. Finally, the breathing pacer was fixed and open-loop. Its physiological effect and participant adherence were not measured. Future studies should compare fixed with individualized or adaptive breathing support, investigate longer and repeated exposure, and determine whether perceived usability, physiological regulation, and control over the NFB signal improve together or represent distinct outcomes [28,30,50].

### Generalizability

The findings are most applicable to brief, first-contact use by self-selected adults in a public, nonclinical exhibition setting. They should not be generalized to clinical populations, repeated home use, long-term adherence, therapeutic NFB, or users requiring substantial assistance with digital technology or EEG hardware without further usability and feasibility testing, in line with recommendations to evaluate sensor-based digital health technologies across relevant user groups and contexts of use [25]. Participation also required the ability to complete the procedure in German or French and willingness to engage with a neurotechnology demonstration in a public exhibition. These requirements may have selected for participants with greater interest in technology or greater confidence in navigating an autonomous tablet-based procedure. Because digital health literacy is closely linked to the adoption and use of digital health technologies [55,56], the present findings may overestimate usability relative to less technology-engaged populations. Nevertheless, the study provides information that may be relevant to similar low-support consumer-EEG applications. In particular, distinguishing session initiation, allocation, contact-quality failure, task completion, posttask exclusion, and final analytic retention offers a useful framework for evaluating autonomous neurotechnology outside controlled laboratory settings. The findings also highlight that the feasibility of the complete user pathway should be evaluated separately from the efficacy of the NFB signal or intervention.

### Conclusion

This study evaluated the complete operation of a fully autonomous, consumer-EEG, beta-responsive visual NFB system in a public first-contact setting. The workflow integrated consent, self-fitting, signal-quality checking, baseline calibration, randomized condition assignment, real-time feedback, and transition to postsession self-report without direct operator assistance. A majority of session initiators completed the NFB task, and participants retained in the analytic sample reported generally favorable usability. However, autonomous completion was not universal, and insufficient EEG contact quality and posttask analytic exclusions substantially reduced the final analytic sample. Adding a fixed, open-loop breathing pacer produced no detectable improvement in usability, subjective relaxation, state mindfulness, or descriptive EEG outcomes, although the trial was not designed to establish equivalence between conditions. Exploratory analyses identified a possible relationship between pretask resting beta activity and perceived usability, particularly perceived functional integration, but the total-score association did not survive correction across the broader EEG-outcome family. The findings therefore provide evidence about autonomous feasibility and usability rather than beta suppression, therapeutic efficacy, or learned self-regulation. Future preregistered studies should retain and validate raw EEG, define progression and feasibility criteria prospectively, assess objective target engagement and breathing adherence, include appropriate control conditions, and examine repeated use. Such work is necessary before autonomous consumer-EEG NFB can be evaluated as a home-based or clinical support tool.

## Supplementary material

10.2196/89256 Multimedia Appendix 1 Short demo video of neurofeedback training with pacer.

10.2196/89256 Multimedia Appendix 2 Methods, analyses, and figures for feasibility and usability study of self-guided, beta-responsive visual neurofeedback using a consumer electroencephalography headband in a public exhibition.

10.2196/89256 Checklist 1 CONSORT 2025 checklist.

10.2196/89256 Checklist 2 CONSORT pilot checklist.

10.2196/89256 Checklist 3 TIDieR checklist.

10.2196/89256 Checklist 4 CONSORT-eHEALTH checklist (V 1.6.1).

## Abbreviations

BCI: brain-computer interface
CONSORT: Consolidated Standards of Reporting Trials
EEG: electroencephalography
MAAS-State: Mindful Attention Awareness Scale–State
MCAR: missing completely at random
NFB: neurofeedback
PTSD: posttraumatic stress disorder
SDK: software development kit
SUS: System Usability Scale
TIDieR: Template for Intervention Description and Replication
VAS: Visual Analog Scale
VN: visual neurofeedback only
VN+BP: visual neurofeedback plus breathing pacer
