# Supplementary material for: Self-Guided Beta-Responsive Visual Neurofeedback in a Public Exhibition: Feasibility and Usability Randomized Controlled rial
Source: JMIR Neurotechnol. 2026 Nov 3;5:e89256. doi: 10.2196/89256 (PMC13741342; doi:10.2196/89256)
Supplement: Supplementary file 1 [file neuro-v5-e89256-s002.pdf]

Multimedia Appendix 2

Supplementary Methods, Analyses, and Figures

All EEG-behavior analyses are exploratory and hypothesis-generating (N=52).

Supplementary methods

The EEG-behavior analyses reported here were performed in Python 3.11 (NumPy, pandas, SciPy, statsmodels; matplotlib for figures) and are fully reproducible from the deposited code. Little's MCAR test was computed in R 4.6.1 (naniar 1.1.0). De-identified data and analysis code are openly available on the Open Science Framework (https://osf.io/zfyd7; DOI 10.17605/OSF.IO/ZFYD7). Raw continuous EEG was never retained by the acquisition application; only the Muse SDK band-power estimates (~10 Hz, log-scaled arbitrary units) were logged, and these are shared in full.

Multiplicity policy

Two families were pre-declared. Family A: baseline absolute band power x subjective outcome (12 tests; Benjamini-Hochberg correction; Table S1). Family B: the 10 System Usability Scale (SUS) items x baseline beta, controlled at the family level by permutation (10,000 permutations of baseline beta; Table S2). No composite EEG-outcome association survives correction (all BH-adjusted P >= .296); only the single item Q5 ("functions well integrated") survives (Holm-adjusted P=.014; maximum-item permutation P=.015). Robustness/sensitivity analyses of the baseline-beta x SUS-total association are summarized in Table S3.

Table S1. EEG–outcome correlation family (Family A)

Baseline absolute band power x subjective outcome. Spearman rho; 95% CI via Fisher z; n=52 (SUS, relaxation), n=51 (mindfulness); Benjamini-Hochberg across 12 tests. No test survives correction.

Predictor x Outcome	rho	95% CI	raw P	BH-adj P
Beta x SUS	-0.311	-0.54, -0.04	.025	.296
Alpha x SUS	-0.199	-0.45, 0.08	.158	.607
Gamma x SUS	-0.191	-0.44, 0.09	.175	.607
Theta x SUS	-0.176	-0.43, 0.11	.212	.607
Gamma x Mindfulness	+0.150	-0.13, 0.41	.292	.607
Gamma x Relaxation	-0.132	-0.39, 0.15	.350	.607
Beta x Relaxation	-0.127	-0.39, 0.15	.369	.607
Beta x Mindfulness	+0.110	-0.17, 0.37	.442	.607
Theta x Mindfulness	+0.096	-0.18, 0.36	.502	.607
Alpha x Relaxation	-0.094	-0.36, 0.18	.506	.607
Theta x Relaxation	-0.045	-0.31, 0.23	.749	.818
Alpha x Mindfulness	+0.017	-0.26, 0.29	.905	.905

Table S2. SUS items x baseline beta (Family B)

Items oriented so that higher scores denote better usability; rho = Spearman with baseline beta (negative = higher beta associated with poorer usability). Family-level control by permutation: global pattern permutation P=.019; maximum-item permutation P=.015 (Q5). Nine of ten items point negative.

SUS item	rho	95% CI	raw P
Q5 functional integration	-0.432	-0.63, -0.18	.001
Q9 confidence	-0.287	-0.52, -0.01	.039
Q1 frequent use	-0.257	-0.49, 0.02	.066
Q8 not cumbersome	-0.238	-0.48, 0.04	.089
Q4 independence	-0.204	-0.45, 0.07	.147
Q2 simplicity	-0.154	-0.41, 0.12	.276
Q10 low learning	-0.119	-0.38, 0.16	.402
Q6 consistency	-0.076	-0.34, 0.20	.592
Q7 learnability	-0.037	-0.31, 0.24	.795
Q3 ease of use	+0.042	-0.23, 0.31	.770

Table S3. Robustness / sensitivity battery

Robustness / sensitivity analyses of the baseline-beta x SUS-total association. All directionally consistent with a negative association.

• Nonparametric bootstrap (10,000): 95% CI -0.54 to -0.05; 98.9% of resamples negative

• Permutation of the total-score correlation (10,000): two-sided P=.026

• Leave-one-out (52 refits): rho in [-0.37, -0.27], sign invariant; 2/52 deletions raise P>=.05

• Outlier-robust (drop |z|>2.5 / 2.5% winsorize): rho = -0.30 to -0.31

• Robust regression, Huber M-estimator (SUS ~ beta + age + sex + condition): b=-13.4, P=.084

• Robust regression, Tukey biweight: b=-13.2, P=.080

• Linear mixed item model (10 items, participant random intercept): b=-0.48, P=.078

• Bayesian correlation (Fisher z, flat prior): posterior median rho=-0.31, 95% CrI -0.54 to -0.04; Pr(rho<0)=0.99

Compatibility: the confidence/credible interval was compatible with a small-to-moderate negative association but remained close to zero at the upper bound, so a near-null effect cannot be excluded.

Table S4. EEG absolute band power by condition

Mean (SD) SDK-derived absolute band power (log-scaled a.u.), averaged over AF7, AF8, TP9, TP10, for the 30-s eyes-closed baseline and 120-s task, by condition. No band differed between conditions at baseline or task (Mann-Whitney U, all P>.05).

Band	Base:Total	Base:VN+BP	Base:VN	Task:Total	Task:VN+BP	Task:VN
Theta	0.39 (0.31)	0.46 (0.24)	0.33 (0.36)	0.34 (0.31)	0.39 (0.23)	0.29 (0.36)
Alpha	0.51 (0.33)	0.56 (0.23)	0.47 (0.39)	0.47 (0.31)	0.53 (0.23)	0.42 (0.37)
Beta	0.36 (0.29)	0.40 (0.20)	0.32 (0.36)	0.36 (0.28)	0.42 (0.20)	0.30 (0.33)
Gamma	0.10 (0.28)	0.13 (0.19)	0.07 (0.34)	0.07 (0.27)	0.12 (0.17)	0.02 (0.34)

Figure S1

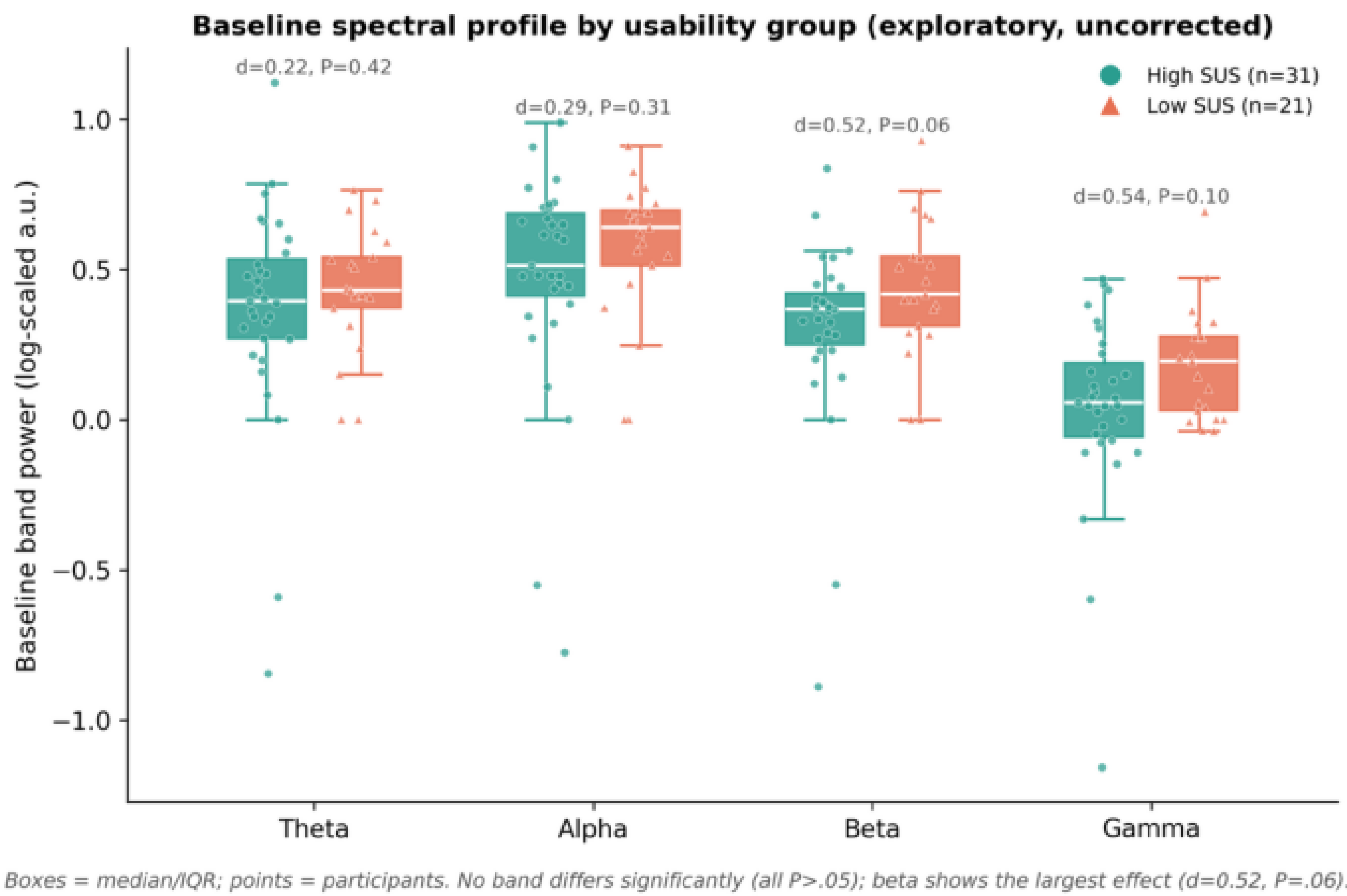

Figure S1. Baseline spectral profile by usability group. Mean baseline absolute band power (log-scaled a.u.) for the SUS >=68 (n=31) and SUS <68 (n=21) groups. Between-group differences were exploratory and non-significant (beta d=0.52, P=.06; gamma d=0.54, P=.10; effect sizes calculated as low vs high usability).

Figure S2

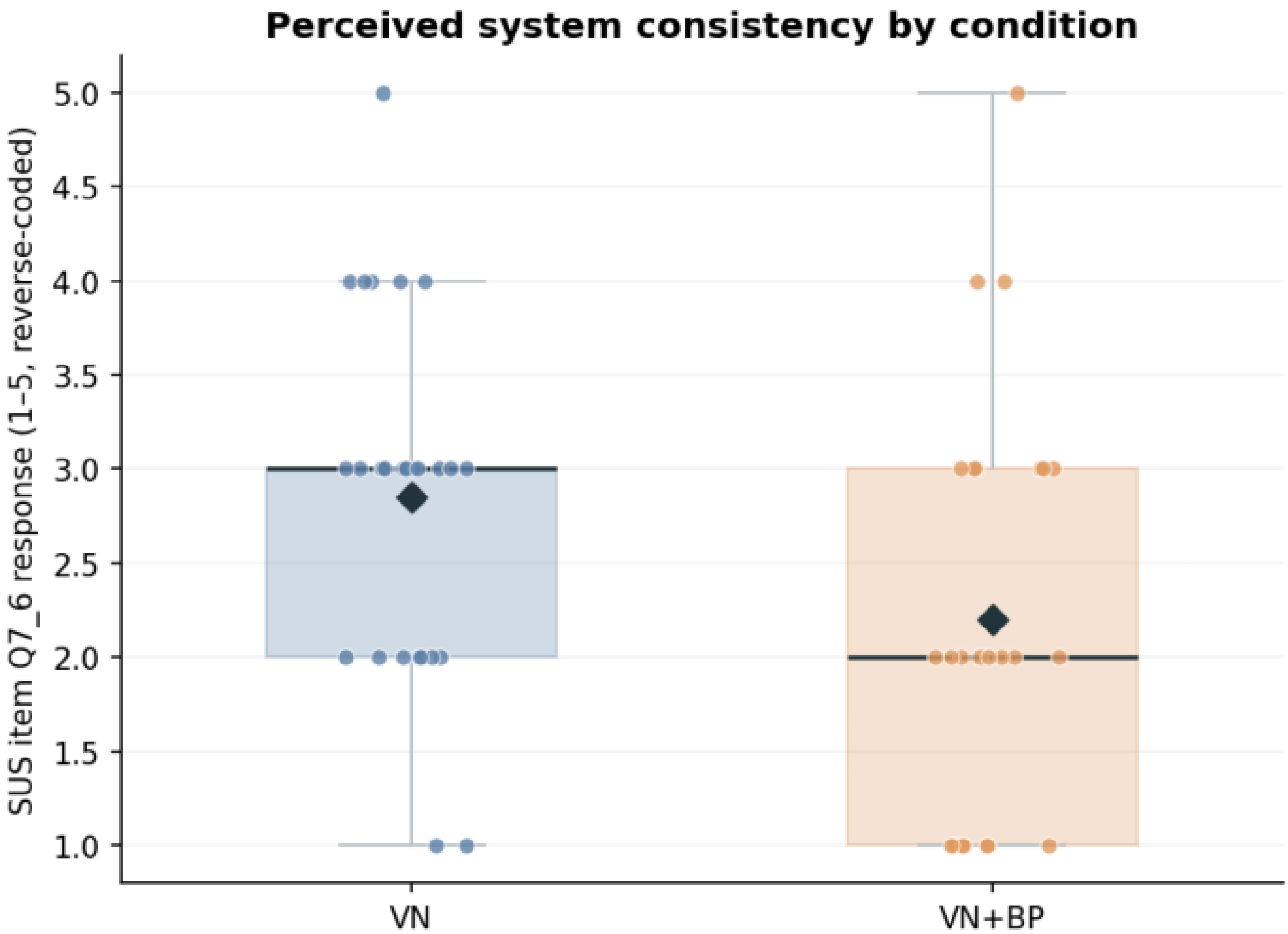

Figure S2. Perceived system consistency (reverse-coded SUS item Q7_6) by condition. Exploratory item-level comparison: the VN+BP condition showed lower raw scores than VN (Mann-Whitney U=459.5, raw P=.02), uncorrected for the 10 SUS items and non-significant after Holm adjustment. Descriptive only; no construct-level or causal interpretation.

Figure S3

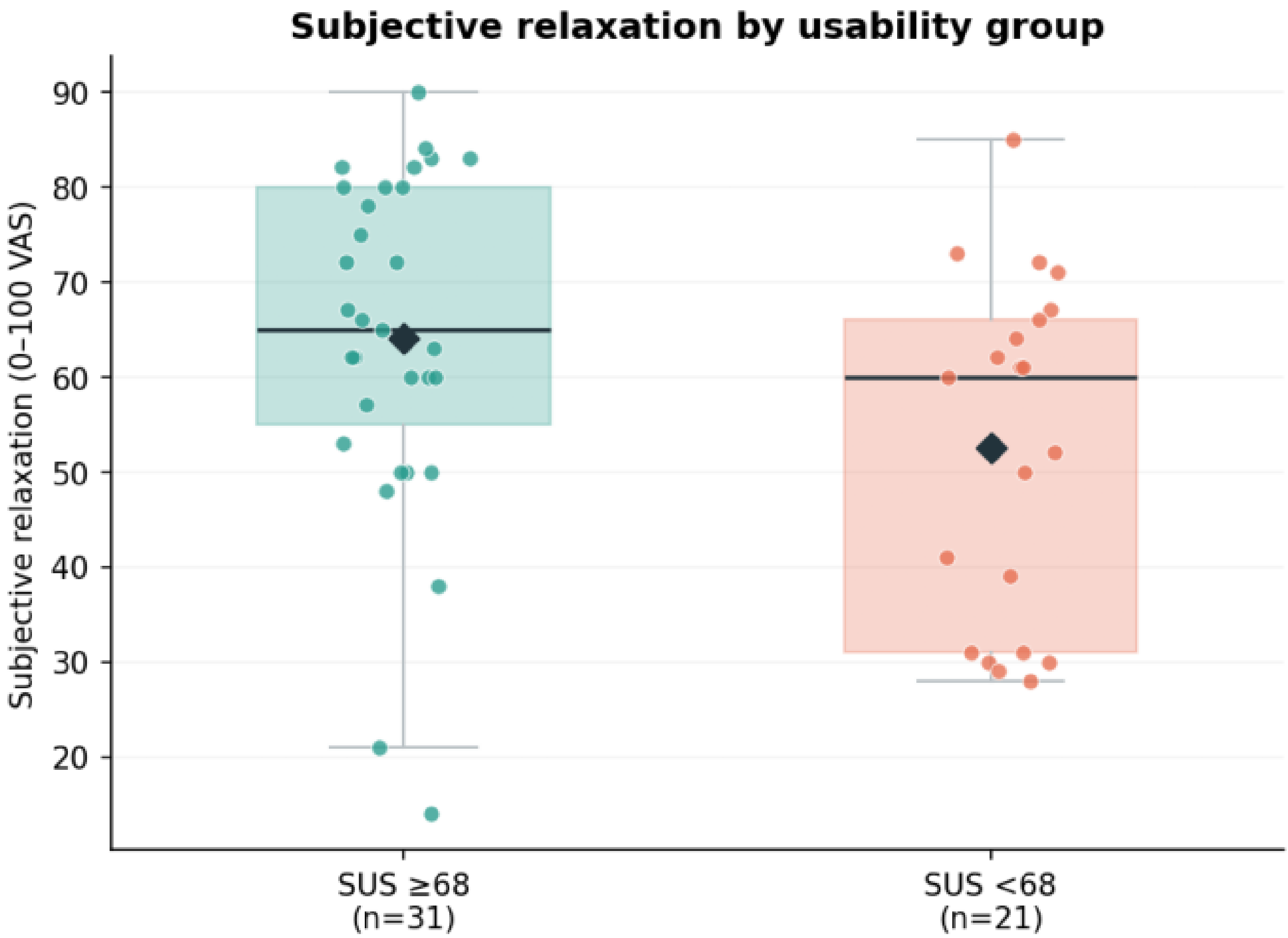

Figure S3. Subjective relaxation (0-100 VAS) by usability group. The SUS >=68 group reported higher relaxation (M=64.1) than the SUS <68 group (M=52.5; Mann-Whitney U=437, raw P=.04; Cohen d=-0.64, 95% CI -1.21 to -0.08, calculated as low vs high usability). Exploratory; does not survive multiplicity correction across the group comparisons (BH-adjusted P=.18).
