# Supplementary material for: Self-Guided Beta-Responsive Visual Neurofeedback in a Public Exhibition: Feasibility and Usability Randomized Controlled rial
Source: JMIR Neurotechnol. 2026 Nov 3;5:e89256. doi: 10.2196/89256 (PMC13741342; doi:10.2196/89256)
Supplement: Supplementary file 2 [file neuro-v5-e89256-s003.pdf]

CONSORT 2025 checklist of information to include when reporting a randomised trial

Manuscript: Self-Guided Beta-Responsive Visual Neurofeedback in a Public Exhibition: A Feasibility and Usability Randomized Controlled Trial

JMIR Neurotechnology manuscript #89256. Page numbers refer to the revised manuscript; section names are given to aid navigation.

Section/Topic	No	CONSORT 2025 checklist item	Where reported
**Title and abstract**			
Title and structured abstract	1a	Identification as a randomised trial	p. 1 (Title)
	1b	Structured summary of the trial design, methods, results, and conclusions	p. 2 (Abstract)
**Open science**			
Trial registration	2	Name of trial registry, identifying number (with URL) and date of registration	p. 13 (Methods: Registration). The trial was not registered in a primary trial registry; study materials, deidentified data, and analysis documentation were deposited in the Open Science Framework.
Protocol and statistical analysis plan	3	Where the trial protocol and statistical analysis plan can be accessed	p. 13 (Methods: Protocol). No prospectively registered protocol or statistical analysis plan was available; available study documentation is on OSF (https://osf.io/zfyd7).
Data sharing	4	Where and how the individual de-identified participant data (including data dictionary), statistical code and any other materials can be accessed	p. 30 (Data Availability)
Funding and conflicts of interest	5a	Sources of funding and other support (eg, supply of drugs), and role of funders in the design, conduct, analysis and reporting of the trial	p. 30 (Funding)
	5b	Financial and other conflicts of interest of the manuscript authors	p. 30 (Conflicts of Interest)
**Introduction**			
Background and rationale	6	Scientific background and rationale	pp. 3–4 (Introduction)
Objectives	7	Specific objectives related to benefits and harms	p. 4 (Introduction: research questions RQ1–RQ3)
**Methods**			
Patient and public involvement	8	Details of patient or public involvement in the design, conduct and reporting of the trial	p. 5 (Methods: Patient and Public Involvement)
Trial design	9	Description of trial design including type of trial (eg, parallel group, crossover), allocation ratio, and framework (eg, superiority, equivalence, non-inferiority, exploratory)	pp. 4–5 (Methods: Trial Design); p. 10 (Sample Size: exploratory feasibility framework)
Changes to trial protocol	10	Important changes to the trial after it commenced including any outcomes or analyses that were not prespecified, with reason	pp. 4–5 (Trial Design: no changes after commencement); pp. 12–13 (Statistical Analyses: exploratory analyses identified as such)
Trial setting	11	Settings (eg, community, hospital) and locations (eg, countries, sites) where the trial was conducted	pp. 4–5 (Trial Design; Participants)

Section/Topic	No	CONSORT 2025 checklist item	Where reported
Eligibility criteria	12a	Eligibility criteria for participants	p. 5 (Participants)
	12b	If applicable, eligibility criteria for sites and for individuals delivering the interventions (eg, surgeons, physiotherapists)	Not applicable: single site; the intervention was delivered by a tablet application without intervention providers (p. 6, Procedure).
Intervention and comparator	13	Intervention and comparator with sufficient details to allow replication. If relevant, where additional materials describing the intervention and comparator (eg, intervention manual) can be accessed	pp. 6–9 (Procedure; Neurofeedback System; EEG Acquisition and Feedback Signal; Experimental Conditions; Intervention Fidelity); Figures 1–2; TIDieR checklist (Multimedia Appendix)
Outcomes	14	Prespecified primary and secondary outcomes, including the specific measurement variable (eg, systolic blood pressure), analysis metric (eg, change from baseline, final value, time to event), method of aggregation (eg, median, proportion), and time point for each outcome	pp. 9–10 (Outcomes)
Harms	15	How harms were defined and assessed (eg, systematically, non-systematically)	p. 24 (Results: Harms). Harms were not assessed systematically; this is stated explicitly.
Sample size	16a	How sample size was determined, including all assumptions supporting the sample size calculation	p. 10 (Sample Size)
	16b	Explanation of any interim analyses and stopping guidelines	Not applicable: no interim analyses or stopping guidelines; the trial ended as planned at the end of the exhibition period (p. 15, Recruitment).
Randomisation: Sequence generation	17a	Who generated the random allocation sequence and the method used	p. 10 (Sequence Generation)
	17b	Type of randomisation and details of any restriction (eg, stratification, blocking and block size)	p. 6 (Procedure: simple randomization without blocking or stratification); p. 10 (Sequence Generation)
Allocation concealment mechanism	18	Mechanism used to implement the random allocation sequence (eg, central computer/telephone; sequentially numbered, opaque, sealed containers), describing any steps to conceal the sequence until interventions were assigned	p. 10 (Allocation Concealment Mechanism)
Implementation	19	Whether the personnel who enrolled and those who assigned participants to the interventions had access to the random allocation sequence	p. 11 (Implementation)
Blinding	20a	Who was blinded after assignment to interventions (eg, participants, care providers, outcome assessors, data analysts)	p. 11 (Blinding)
	20b	If blinded, how blinding was achieved and description of the similarity of interventions	pp. 8–9 (Experimental Conditions); p. 11 (Blinding). Participants could not be blinded to the breathing pacer.
Statistical methods	21a	Statistical methods used to compare groups for primary and secondary outcomes, including harms	pp. 12–13 (Statistical Analyses)
	21b	Definition of who is included in each analysis (eg, all randomised participants), and in which group	p. 11 (Analysis Populations and Exclusion Criteria)
	21c	How missing data were handled in the analysis	p. 12 (Missing Data)

Section/Topic	No	CONSORT 2025 checklist item	Where reported
	21d	Methods for any additional analyses (eg, subgroup and sensitivity analyses), distinguishing prespecified from post hoc	pp. 12–13 (Statistical Analyses); Multimedia Appendix 1
**Results**			
Participant flow, including flow diagram	22a	For each group, the numbers of participants who were randomly assigned, received intended intervention, and were analysed for the primary outcome	p. 14 (Participant Flow); Figure 3 (CONSORT 2025 flow diagram)
	22b	For each group, losses and exclusions after randomisation, together with reasons	p. 14 (Participant Flow); Figure 3
Recruitment	23a	Dates defining the periods of recruitment and follow-up for outcomes of benefits and harms	p. 15 (Recruitment); single session without follow-up beyond the post-session questionnaire
	23b	If relevant, why the trial ended or was stopped	p. 15 (Recruitment: ended as planned at the conclusion of the exhibition period)
Intervention and comparator delivery	24a	Intervention and comparator as they were actually administered (eg, where appropriate, who delivered the intervention/comparator, whether participants adhered, whether they were delivered as intended [fidelity])	p. 9 (Intervention Fidelity); p. 14 (Participant Flow); pp. 16–17 (Table 1; Feasibility of Autonomous Use). Adherence to the breathing pacer was not measured (p. 29, Limitations).
	24b	Concomitant care received during the trial for each group	Not applicable: single 10–12-minute session without concomitant care. Recent alcohol or stimulant medication use was recorded and used as an analytic exclusion criterion (p. 11; p. 14).
Baseline data	25	A table showing baseline demographic and clinical characteristics for each group	pp. 15–16 (Baseline Data; Table 1)
Numbers analysed, outcomes and estimation	26	For each primary and secondary outcome, by group: the number of participants included in the analysis; the number of participants with available data at the outcome time point; result for each group, and the estimated effect size and its precision (such as 95% confidence interval); for binary outcomes, presentation of both absolute and relative effect size	p. 17 (Numbers Analyzed); pp. 17–20 (Psychological Outcomes by Randomized Condition; Table 2; Figure 4; Descriptive SDK-Derived EEG Band-Power Estimates; Table 3). The binary SUS ≥68 outcome is reported descriptively with counts and proportions by group.
Harms	27	All harms or unintended events in each group	p. 24 (Harms). Harms were not assessed systematically; no adverse events were reported, which is not interpreted as evidence of their absence.
Ancillary analyses	28	Any other analyses performed, including subgroup and sensitivity analyses, distinguishing prespecified from post hoc	pp. 20–23 (Ancillary Analyses; Table 4; Figures 5–6); Multimedia Appendix 1. All ancillary analyses are labeled exploratory.
**Discussion**			
Interpretation	29	Interpretation consistent with results, balancing benefits and harms, and considering other relevant evidence	pp. 24–28 (Discussion)
Limitations	30	Trial limitations, addressing sources of potential bias, imprecision, generalisability, and, if relevant, multiplicity of analyses	pp. 28–29 (Limitations; Generalizability)

Citation: Hopewell S, Chan AW, Collins GS, Hróbjartsson A, Moher D, Schulz KF, et al. CONSORT 2025 statement: updated guideline for reporting randomised trials. BMJ. 2025;389:e081123. The checklist is distributed under the Creative Commons Attribution License (CC BY 4.0).
