# Supplementary material for: Self-Guided Beta-Responsive Visual Neurofeedback in a Public Exhibition: Feasibility and Usability Randomized Controlled rial
Source: JMIR Neurotechnol. 2026 Nov 3;5:e89256. doi: 10.2196/89256 (PMC13741342; doi:10.2196/89256)
Supplement: Supplementary file 3 [file neuro-v5-e89256-s004.pdf]

CONSORT 2010 checklist of information to include when reporting a pilot or feasibility trial

Manuscript: Self-Guided Beta-Responsive Visual Neurofeedback in a Public Exhibition: A Feasibility and Usability Randomized Controlled Trial

JMIR Neurotechnology manuscript #89256. Page numbers refer to the revised manuscript; section names are given to aid navigation.

Section/Topic	Item No	Checklist item	Reported on page No
**Title and abstract**			
	1a	Identification as a pilot or feasibility randomised trial in the title	p. 1 (Title)
	1b	Structured summary of pilot trial design, methods, results, and conclusions (for specific guidance see CONSORT abstract extension for pilot trials)	p. 2 (Abstract)
**Introduction**			
Background and objectives	2a	Scientific background and explanation of rationale for future definitive trial, and reasons for randomised pilot trial	pp. 3–4 (Introduction)
	2b	Specific objectives or research questions for pilot trial	p. 4 (RQ1–RQ3)
**Methods**			
Trial design	3a	Description of pilot trial design (such as parallel, factorial) including allocation ratio	pp. 4–5 (Trial Design)
	3b	Important changes to methods after pilot trial commencement (such as eligibility criteria), with reasons	pp. 4–5 (Trial Design: no changes after trial commencement)
Participants	4a	Eligibility criteria for participants	p. 5 (Participants)
	4b	Settings and locations where the data were collected	pp. 4–5 (Trial Design; Participants)
	4c	How participants were identified and consented	p. 5 (Participants); p. 13 (Ethical Considerations)
Interventions	5	The interventions for each group with sufficient details to allow replication, including how and when they were actually administered	pp. 6–9 (Procedure; Neurofeedback System; EEG Acquisition and Feedback Signal; Experimental Conditions; Intervention Fidelity); Figures 1–2
Outcomes	6a	Completely defined prespecified assessments or measurements to address each pilot trial objective specified in 2b, including how and when they were assessed	pp. 9–10 (Outcomes)
	6b	Any changes to pilot trial assessments or measurements after the pilot trial commenced, with reasons	pp. 4–5 (Trial Design: no changes after trial commencement)
	6c	If applicable, prespecified criteria used to judge whether, or how, to proceed with future definitive trial	p. 10 (Outcomes) and p. 28 (Discussion): no formal progression criteria were prespecified
Sample size	7a	Rationale for numbers in the pilot trial	p. 10 (Sample Size)
	7b	When applicable, explanation of any interim analyses and stopping guidelines	Not applicable: no interim analyses or stopping guidelines
Randomisation: Sequence generation	8a	Method used to generate the random allocation sequence	p. 10 (Sequence Generation)

Section/Topic	Item No	Checklist item	Reported on page No
	8b	Type of randomisation(s); details of any restriction (such as blocking and block size)	p. 6 (Procedure); p. 10 (Sequence Generation)
Allocation concealment mechanism	9	Mechanism used to implement the random allocation sequence (such as sequentially numbered containers), describing any steps taken to conceal the sequence until interventions were assigned	p. 10 (Allocation Concealment Mechanism)
Implementation	10	Who generated the random allocation sequence, who enrolled participants, and who assigned participants to interventions	p. 11 (Implementation)
Blinding	11a	If done, who was blinded after assignment to interventions (for example, participants, care providers, those assessing outcomes) and how	p. 11 (Blinding)
	11b	If relevant, description of the similarity of interventions	pp. 8–9 (Experimental Conditions); p. 11 (Blinding)
Statistical methods	12	Methods used to address each pilot trial objective whether qualitative or quantitative	pp. 11–13 (Statistical Methods: Analysis Populations; Missing Data; Statistical Analyses)
**Results**			
Participant flow (a diagram is strongly recommended)	13a	For each group, the numbers of participants who were approached and/or assessed for eligibility, randomly assigned, received intended treatment, and were assessed for each objective	p. 14 (Participant Flow); Figure 3
	13b	For each group, losses and exclusions after randomisation, together with reasons	p. 14 (Participant Flow); Figure 3
Recruitment	14a	Dates defining the periods of recruitment and follow-up	p. 15 (Recruitment)
	14b	Why the pilot trial ended or was stopped	p. 15 (Recruitment)
Baseline data	15	A table showing baseline demographic and clinical characteristics for each group	pp. 15–16 (Baseline Data; Table 1)
Numbers analysed	16	For each objective, number of participants (denominator) included in each analysis. If relevant, these numbers should be by randomised group	p. 17 (Numbers Analyzed)
Outcomes and estimation	17	For each objective, results including expressions of uncertainty (such as 95% confidence interval) for any estimates. If relevant, these results should be by randomised group	pp. 14 and 17–20 (Participant Flow; Feasibility of Autonomous Use; Psychological Outcomes; Tables 2–3; Figure 4)
Ancillary analyses	18	Results of any other analyses performed that could be used to inform the future definitive trial	pp. 20–23 (Ancillary Analyses; Table 4; Figures 5–6); Multimedia Appendix 1
Harms	19	All important harms or unintended effects in each group (for specific guidance see CONSORT for harms)	p. 24 (Harms)
	19a	If relevant, other important unintended consequences	p. 24 (Harms: not systematically assessed); p. 14 (17 sessions with insufficient EEG contact quality)
**Discussion**			
Limitations	20	Pilot trial limitations, addressing sources of potential bias and	pp. 28–29 (Limitations)

Section/Topic	Item No	Checklist item	Reported on page No
		remaining uncertainty about feasibility	
Generalisability	21	Generalisability (applicability) of pilot trial methods and findings to future definitive trial and other studies	p. 29 (Generalizability)
Interpretation	22	Interpretation consistent with pilot trial objectives and findings, balancing potential benefits and harms, and considering other relevant evidence	pp. 24–28 (Discussion)
	22a	Implications for progression from pilot to future definitive trial, including any proposed amendments	p. 28 (Exploratory Resting Beta-Usability Association, final paragraph); p. 29 (Conclusion)
**Other information**			
Registration	23	Registration number for pilot trial and name of trial registry	p. 13 (Registration: not registered in a primary trial registry)
Protocol	24	Where the pilot trial protocol can be accessed, if available	p. 13 (Protocol: no prospectively registered protocol; study documentation on OSF)
Funding	25	Sources of funding and other support (such as supply of drugs), role of funders	p. 30 (Funding)
	26	Ethical approval or approval by research review committee, confirmed with reference number	p. 13 (Ethical Considerations: ETH Zurich Ethics Commission, 25 ETHICS-086)

Citation: Eldridge SM, Chan CL, Campbell MJ, Bond CM, Hopewell S, Thabane L, et al. CONSORT 2010 statement: extension to randomised pilot and feasibility trials. BMJ. 2016;355:i5239. Open Access (CC BY 3.0).
