# Supplementary material for: Self-Guided Beta-Responsive Visual Neurofeedback in a Public Exhibition: Feasibility and Usability Randomized Controlled rial
Source: JMIR Neurotechnol. 2026 Nov 3;5:e89256. doi: 10.2196/89256 (PMC13741342; doi:10.2196/89256)
Supplement: Supplementary file 4 [file neuro-v5-e89256-s005.pdf]

The TIDieR (Template for Intervention Description and Replication) Checklist*:

Information to include when describing an intervention and the location of the information

Manuscript: Self-Guided Beta-Responsive Visual Neurofeedback in a Public Exhibition: A Feasibility and Usability Randomized Controlled Trial (JMIR Neurotechnology manuscript #89256)

Item number	Item	Where located** Primary paper (page or appendix number)	Where located** Other† (details)
	**BRIEF NAME**		
1.	Provide the name or a phrase that describes the intervention.	p. 1 (Title); p. 4 (Introduction)	Visual neurofeedback only (VN) vs visual neurofeedback plus breathing pacer (VN+BP)
	**WHY**		
2.	Describe any rationale, theory, or goal of the elements essential to the intervention.	pp. 3–4 (Introduction)	
	**WHAT**		
3.	**Materials:** Describe any physical or informational materials used in the intervention, including those provided to participants or used in intervention delivery or in training of intervention providers. Provide information on where the materials can be accessed (e.g. online appendix, URL).	pp. 6–8 (Procedure; Neurofeedback System; EEG Acquisition and Feedback Signal); Figures 1–2	Study documentation: OSF, https://osf.io/zfyd7 (p. 30); the application is not publicly distributed
4.	**Procedures:** Describe each of the procedures, activities, and/or processes used in the intervention, including any enabling or support activities.	pp. 6–9 (Procedure; EEG Acquisition and Feedback Signal; Experimental Conditions); Figure 1	
	**WHO PROVIDED**		
5.	For each category of intervention provider (e.g. psychologist, nursing assistant), describe their expertise, background and any specific training given.	p. 6 (Procedure); p. 11 (Implementation; Blinding)	N/A: no human intervention provider; delivery was fully automated by the tablet application
	**HOW**		
6.	Describe the modes of delivery (e.g. face-to-face or by some other mechanism, such as internet or telephone) of the intervention and whether it was provided individually or in a group.	p. 6 (Procedure); p. 7 (Neurofeedback System)	
	**WHERE**		
7.	Describe the type(s) of location(s) where the intervention occurred, including any necessary infrastructure or relevant features.	pp. 4–5 (Trial Design; Participants); p. 7 (Neurofeedback System)	
	**WHEN and HOW MUCH**		
8.	Describe the number of times the intervention was delivered and over what period of time including the number of sessions, their schedule, and their duration, intensity or dose.	p. 6 (Procedure); p. 9 (Intervention Fidelity); p. 15 (Recruitment)	
	**TAILORING**		
9.	If the intervention was planned to be personalised, titrated or adapted, then describe what, why, when, and how.	p. 8 (EEG Acquisition and Feedback Signal); pp. 8–9 (Experimental Conditions); p. 9 (Intervention Fidelity)	
	**MODIFICATIONS**		
10.ǂ	If the intervention was modified during the course of the study,	pp. 4–5 (Trial Design:	

Item number	Item	Where located** Primary paper (page or appendix number)	Where located** Other† (details)
	describe the changes (what, why, when, and how).	no modifications after trial commencement)	
	**HOW WELL**		
11.	**Planned:** If intervention adherence or fidelity was assessed, describe how and by whom, and if any strategies were used to maintain or improve fidelity, describe them.	p. 7 (Neurofeedback System); p. 9 (Intervention Fidelity)	
12.ǂ	**Actual:** If intervention adherence or fidelity was assessed, describe the extent to which the intervention was delivered as planned.	p. 14 (Participant Flow); Figure 3; pp. 16–17 (Table 1; Feasibility of Autonomous Use); p. 29 (Limitations)	

** Authors – use N/A if an item is not applicable for the intervention being described. Reviewers – use '?' if information about the element is not reported/not sufficiently reported.

† If the information is not provided in the primary paper, give details of where this information is available. This may include locations such as a published protocol or other published papers (provide citation details) or a website (provide the URL).

ǂ If completing the TIDieR checklist for a protocol, these items are not relevant to the protocol and cannot be described until the study is complete.

* We strongly recommend using this checklist in conjunction with the TIDieR guide (see BMJ 2014;348:g1687) which contains an explanation and elaboration for each item.

* The focus of TIDieR is on reporting details of the intervention elements (and where relevant, comparison elements) of a study. Other elements and methodological features of studies are covered by other reporting statements and checklists and have not been duplicated as part of the TIDieR checklist. When a randomised trial is being reported, the TIDieR checklist should be used in conjunction with the CONSORT statement (see www.consort-statement.org) as an extension of Item 5 of the CONSORT 2010 Statement.

Citation: Hoffmann TC, Glasziou PP, Boutron I, et al. Better reporting of interventions: template for intervention description and replication (TIDieR) checklist and guide. BMJ. 2014;348:g1687. Page numbers refer to the revised manuscript.
